# Reforming the police through procedural justice training: A multicity randomized trial at crime hot spots

**DOI:** 10.1073/pnas.2118780119

**Published:** 2022-03-28

**Authors:** David Weisburd, Cody W. Telep, Heather Vovak, Taryn Zastrow, Anthony A. Braga, Brandon Turchan

**Affiliations:** ^a^Department of Criminology, Law and Society, George Mason University, Fairfax, VA 22030;; ^b^Institute of Criminology, The Hebrew University of Jerusalem, Jerusalem 91905, Israel;; ^c^School of Criminology and Criminal Justice, Arizona State University, Phoenix, AZ 85004;; ^d^National Policing Institute, Arlington, VA 22202;; ^e^Department of Criminology, University of Pennsylvania, Philadelphia, PA 19104

**Keywords:** procedural justice, police training, hot spots policing, randomized controlled trial

## Abstract

Can police be trained to treat people in fair and respectful ways, and if so, will this influence evaluations of the police and crime? To answer these questions, we randomly allocated 120 crime hot spots to a procedural justice (PJ) and standard condition (SC) in three cities. Twenty-eight officers were randomly assigned to the conditions. The PJ condition officers received an intensive 5-d training course in the components of PJ (giving voice, showing neutrality, treating people with respect, and evidencing trustworthy motives). We used police self-report surveys to assess whether the training influenced attitudes, systematic social observations to examine impacts on police behavior in the field, and arrests to assess law enforcement actions. We conducted pre and post household surveys to assess resident attitudes toward the police. Impacts on crime were measured using crime incident and citizen-initiated crime call data. The training led to increased knowledge about PJ and more procedurally just behavior in the field as compared with the SC condition. At the same time, PJ officers made many fewer arrests than SC officers. Residents of the PJ hot spots were significantly less likely to perceive police as harassing or using unnecessary force, though we did not find significant differences between the PJ and SC hot spots in perceptions of PJ and police legitimacy. We found a significant relative 14% decline in crime incidents in the PJ hot spots during the experiment.

## Significance

Our study is a randomized trial in policing confirming that intensive training in procedural justice (PJ) can lead to more procedurally just behavior and less disrespectful treatment of people at high-crime places. The fact that the PJ intervention reduced arrests by police officers, positively influenced residents’ perceptions of police harassment and violence, and also reduced crime provides important guidance for police reform in a period of strong criticism of policing. This randomized trial points to the potential for PJ training not simply to encourage fair and respectful policing but also to improve evaluations of the police and crime prevention effectiveness.

There has been a growing call for reform of the police (1, 2), often in response to social media documentation of police disrespect, harassment, and violence (3). Criticism of “how the police police” has been particularly strong in critiques of proactive policing strategies. There is now substantial evidence that proactive policing can have meaningful effects on crime, especially when it is focused at crime hot spots—small areas, such as street segments that produce a substantial part of the crime problem (4–6). However, there is at the same time strong concern that proactive policing at crime hot spots may lead to increased police abuses and negative community evaluations of the police (7, 8).

Procedural justice (PJ) involves fair and respectful treatment of people by police (giving voice, showing neutrality, treating people with dignity and respect, and evidencing trustworthy motives) (9). Scholars have predicted that if the police behave in procedurally just ways they will not only improve peoples’ evaluations of police but they will also reduce crime (10, 11). However, the National Academy of Sciences Committee on Proactive Policing concluded in 2018 that there were simply not enough rigorous empirical studies to draw conclusions regarding the impacts of PJ policing on attitudes or its ability to reduce crime (4). Despite a growing number of studies of PJ in policing since that time (12–14), there remain few randomized field trials from which to draw concrete conclusions, and none that focus on crime hot spots (15).

In this paper, we present findings from a three-city (Tucson, AZ; Cambridge, MA; and Houston, TX) randomized field trial that tested whether PJ training would impact police officer behavior, hot-spot residents’ perceptions of police, and crime. The intervention period was 9 mo in each city. We also began the study in a fourth city, but COVID-19 led to implementation of treatment for only half of the study period and prevented key data collection (*SI Appendix*, section S1). We first carried out the experiment in Tucson (1 July 2017 to 31 March 2018) as a test of whether the training and treatment could be delivered as intended. We then moved to Cambridge (2 February 2019 to 31 October 2019) and Houston (12 October 2019 to 10 July 2020). We compare two conditions, PJ hot spots patrolled by officers that received training (see *Selection, Allocation, and Training of Patrol Officers*) and were encouraged to build trust and reduce crime in hot spots and standard-condition (SC) hot spots where officers focused just on reducing crime.

## Methods

The focus of the intervention was on crime hot spots, which were defined as high-crime residential street segments, intersection to intersection. We used crime incident data, which represent crimes reported by the police, and citizen-initiated crime calls to identify hot spots (*SI Appendix*, section S1.1 and Tables S1–S3). We required that the hot spots had at least 15 residential dwelling units to ensure a sufficient sampling frame for our community survey. We selected 40 crime hot spots in each city at least one street segment distant from each other. A power analysis shows a minimal detectable standardized effect size of 0.23 for nondirectional tests and 0.20 for directional tests, suggesting that the study was sufficiently powered to identify small to moderate program impacts for outcomes at the hot-spot level (*SI Appendix*, section S1.2).

After selecting the crime hot spots, they were randomly assigned, within statistical blocks defined by crime levels, equally to the PJ and SC groups (*SI Appendix*, section S1.2 and Tables S4–S6). In the selection year, the sample hot spots in Tucson had a mean of 133.98 crime incidents (median = 120.5, SD = 61.74) and 216.02 citizen-initiated crime calls (median = 189.5, SD = 113.47); Cambridge hot spots had a mean of 17.38 crime incidents (median = 13, SD = 14.79) and 68.73 citizen-initiated crime calls (median = 47.5, SD = 93.4); and Houston hot spots had a mean of 27.78 crime incidents (median = 18.5, SD = 20.57), and 85.8 citizen-initiated crime calls (median = 68.5, SD = 59.6). The demographic characteristics of the hot spots based on the community survey (see *Community Survey*) are reported in *SI Appendix*, Table S7. The respondents in our community survey of the crime hot spots were 40% Latino in Tucson, a majority white in Cambridge, and a majority Black in Houston.

### Selection, Allocation, and Training of Patrol Officers.

In Tucson and Houston, eight officers participated in the program, and in Cambridge 12 officers participated. All officers worked in a patrol role prior to the project start. Once we received a list of the officers and their characteristics, we matched officers in pairs in terms of background, including race and ethnicity, gender, and experience. We then randomly allocated one officer in each pair to the PJ group and one to the SC group. There were no large or statistically significant differences in background characteristics or views of PJ between the groups at baseline (*SI Appendix*, section S1.3 and Tables S8 and S9).

Guided by existing PJ training models, we developed an intensive training program for the PJ officers that was delivered over 40 h (refs. 16–20; *SI Appendix*, Fig. S1). The first day of training included an overview of hot-spots policing, police legitimacy, and PJ. Day two focused on each of the four elements of PJ (voice, neutrality, dignity and respect, and trustworthy motives), as well as the importance of historical context in understanding trust in police. Day three included discussions of scenarios as well as a role-playing exercise where officers had a chance to practice using PJ in interactions. Day four applied the ideas of PJ to working with diverse populations and those with behavioral health problems. The second half of day four introduced the project hot spots and covered applying PJ to hot-spots policing tactics. Finally, day five incorporated plans for officer supervision and officer forms (including an activity log; *SI Appendix*, Fig. S2). The training protocol is detailed in *SI Appendix*, section S1.3. We also followed up with a 1-h, in-person refresher training for PJ officers in each city about 3 mo after the initial training. An officer survey conducted before and after training suggests that officer knowledge of components of PJ significantly improved after the training (*SI Appendix*, sections S1.4 and S2.1 and Table S10). The SC group received a half-day training on hot-spots policing tactics and project data collection, which was also part of the training of the experimental condition.

### Treatment Dosage.

Treatment dosage was measured using activity logs describing the amount of time the study police officers spent at the project sites (*SI Appendix*, section S1.5 and Table S11). In Tucson the PJ hot spots received about 123,911 min of individual police presence, in Cambridge about 136,673 min, and in Houston (where there were often long distances between the hot spots) about 62,974 min. Looking at the SC sites, we gained similar estimates (*SI Appendix*, Tables S12 and S13). While the project officers responded to citizen-initiated calls for service at their assigned hot spots, they spent a substantial amount of time focusing on proactive police activities.

Because the hot spots continued to receive patrol response to emergency calls when project officers were not present, we sought to identify the proportion of overall policing at the PJ hot spots that was provided by PJ officers. To estimate this, we used police management systems, which track police responses to calls for service and officer-initiated activities. The Cambridge data identified police officer minutes directly by officer. Houston provided similar information, but only one officer was listed for each event. Tucson did not include the number of minutes officers spent on site—this was calculated based on data provided by the police department for project officers in the 6 mo preintervention. We estimate that 50.56% of total police time at the hot spots came from experimental officers in Cambridge, 61.23% in Houston, and 48.50% in Tucson (*SI Appendix*, Table S14). While these estimates rely on available data, they suggest that a substantial part of policing in the PJ group hot spots was provided by the project officers. Outcomes are similar for the SC group officers (*SI Appendix*, Table S15).

## Data and Analytic Approach

We used systematic social observations (SSOs) to assess whether the police officers acted in procedurally just ways, arrests as a measure of law enforcement activity, community survey data to assess perceptions of the police, and crime incidents and citizen-initiated crime calls to examine impacts on crime. This project was approved by institutional review boards at Arizona State University and Northeastern University. In order to provide a comparative review of study outcomes, we computed Cohen’s *d* standardized effect sizes for outcome measures, except in the case of arrest and crime outcomes, where Cohen’s *d* cannot be calculated reliably from our statistical analyses. In these cases, we calculate incidence rate ratios (IRR), which provide an easily interpreted metric for understanding the magnitude of study findings. Cohen provided standard metrics for small (*d* = 0.20), moderate (*d* = 0.50), and large effect (*d* = 0.80) sizes, though reviews in criminology suggest that these metrics may be overly conservative (21, 22).

### SSOs.

Trained observers (*SI Appendix*, Fig. S3) were assigned by the research team to ride with one officer for an entire shift, varying which officers rode with which observers and the days of the week each officer was observed (23). Officers provided informed consent prior to the start of the ride. In order to prevent selection biases, we did not consult police managers or observers in our allocation of observers to officers, and observers were blinded as to whether the officers were in the PJ or SC group (*SI Appendix*, section S1.6). We conducted 129 ride-alongs in the three cities (66 in the PJ group and 63 in the SC group). PJ and SC ride-alongs were balanced in the days of the week they were conducted, the officers who were observed, and the number of shifts per officer observed (*SI Appendix*, section S1.6). The ride-alongs yielded 334 encounters with 508 interactions between police officers and citizens. The encounters did not always occur at the hot spots—for example, officers sometimes followed cars outside the hot spots or made an on-site stop of suspicious persons or vehicles riding to or from the hot spots (*SI Appendix*, Table S16). While we think that our assessment of PJ should be based on all officer contacts, we also analyzed data only for the hot spots as a sensitivity analysis.

In analyzing these data, we used a mixed-effects regression modeling approach in which city and treatment group are fixed effects and police officer and encounter are random effects (i.e., a random intercepts model). The model may be expressed as follows where *y* is a component of PJ or an overall procedure justice score, *Group* refers to officer group (PJ group = 1 in all models), *City* is a dummy variable for city, and citizen interactions with police are nested within encounters (*u_j_*) nested within officers (*r_k_*):

$$y_{\mathrm{ijk}}=B_{0}+B_{1}G\mathrm{roup}+B_{2-3}C\mathrm{ity}+r_{k}+u_{j}+e_{i}.$$

From the coding of interactions (*SI Appendix*, Table S17), we produced an overall PJ score and indices for the four main PJ components: voice, neutrality, dignity and respect, and trustworthy motives. Imputed values were generated for voice missing scores when creating the overall PJ measure because in some encounters citizens chose not to respond to officers, so observers were unable to assess whether the officer was an active listener during these encounters (*SI Appendix*, section S1.7). Because disrespectful encounters have been found to be particularly influential on perceptions of police (24, 25), we also measured officer disrespect. These indices were drawn from a prior SSO study that identified formative indices of PJ (26). In contrast to reflective indices, which combine similar variables of the same construct (and are usually assessed for consistency using Cronbach’s α), formative indices bring together distinct items reflecting different elements of a single construct (27, 28). The indices are detailed in *SI Appendix*, section S1.7 and descriptives are in *SI Appendix*, Table S18. We employed one-tailed tests of statistical significance as the treatment has a strong presumption of increasing PJ and reducing police disrespect.

### Arrests.

We collected data on arrests made by project officers in the 6 mo prior to the intervention and during the intervention period (*SI Appendix*, section S2.4 and Table S19). We analyzed these data at the police officer level including treatment group, city, and log of preintervention arrests as independent variables. We model arrest outcomes using the standard log-link function for a negative binomial regression (because of overdispersion in the intervention period) where *y* represents arrests during the intervention period, *Group* refers to officer group (PJ group =1), *City* is a dummy variable for city, and *PreArrests* is an indicator of the number of arrests an officer made in the 6 mo prior to the start of the intervention:

$$\text{ln}(y_{i})=B_{0}+B_{1}G\mathrm{roup}+B_{2-3}C\mathrm{ity}+B_{4}\text{log}\left(\mathrm{PreArrests}\right)+e_{i}.$$

We employed a two-tailed test of statistical significance because there is not a strong prior hypothesis regarding the impacts of PJ training on arrest behavior.

### Community Survey.

The first step in the community survey was to develop a census of households on each street (*SI Appendix*, Fig. S4), from which dwelling units were randomly sampled with the goal of gaining seven surveys per hot spot (*SI Appendix*, Fig. S5). Door-to-door residential surveys were conducted before and after the intervention period. Trained field researchers, who were not told whether they were in a PJ or SC hot spot, interviewed the first adult resident contacted at a household who had lived on the street for at least 3 mo. The surveys were conducted face-to-face with field researchers reading the survey to participants after obtaining informed consent. Respondents were compensated $20 for their participation. The response rate (number of surveys completed/number of households sampled) was 33.53% for the preintervention survey across the three cities. An average of seven surveys were completed on each street in the preintervention survey. Additional details regarding the survey methodology are provided in *SI Appendix*, section S1.8 and Fig. S6.

Data collection in the postintervention survey in Tucson and Cambridge followed protocols similar to the preintervention survey, with the difference that we began sampling with preintervention respondents. For the postintervention survey, the response rate in Tucson and Cambridge was still high at 35.84%. Because of COVID-19, we were not able to carry out door-to-door surveying postintervention in Houston and relied on telephone surveying (*SI Appendix*, Fig. S7). We used contact information provided in the preintervention survey, as well as mailing postcards to sampled households, and purchasing phone numbers from third-party providers (*SI Appendix*, section S1.8). These methods yielded a much lower response rate than the preintervention survey (17.01%). We did not find significant differences in background characteristics between the pre- and postintervention surveys in Houston for gender, race, age, having a college degree, or household size, though postintervention respondents were significantly more likely to own their homes (59.6% versus 46.2%)—reflecting the increased likelihood of identifying phone numbers for homeowners (ref. 29; *SI Appendix*, Table S20). While we were able to collect between 229 and 329 surveys pre and post for Tucson and Cambridge and 277 surveys preintervention in Houston, we were only able to collect 109 surveys postintervention in Houston.

We used a fixed effects ANOVA model to examine change in average perceptions on hot spots comparing the difference between post and preintervention means. In the models estimated, *y* is one of the five survey outcomes examined, *Group* refers to treatment group the hot spot was assigned to, *City* is a dummy variable for city, and *Block* is a dummy variable for statistical block used for hot spot randomization:

$$y_{i}=B_{0}+B_{1}G\mathrm{roup}+B_{2-3}C\mathrm{ity}+B_{4-11}B\mathrm{lock}+e_{i}.$$

As a sensitivity test, we also analyzed data at the respondent level and estimated multilevel mixed-effects linear models with probability weights (*SI Appendix*, section S2.5). We also carried out the analyses involving single response measures using multilevel mixed-effects ordered logistic regression models (*SI Appendix*, section S2.5).

The process model of PJ proposed by scholars assumes that when the police behave in more procedurally just ways people will respond by assessing police authority as more legitimate (9, 10, 30). Our perceptual scale of PJ included 12 questions related to voice, neutrality, dignity and respect, and trustworthy motives (*SI Appendix*, section S1.9 and Table S21; Cronbach’s α = 0.92). Police legitimacy was measured both in terms of attitudes toward police on the street where the respondent lived (*SI Appendix*, Table S22; six items, α = 0.77), as well as for the city overall (*SI Appendix*, Table S23; five items, α = 0.92). All questions used four-item Likert scales (strongly disagree to strongly agree) where higher values indicate more positive views about police.

We also asked residents about their perceptions of police in regard to two issues that have received prominence in recent criticisms of policing. The first asked whether the respondent believed that the police harass or mistreat people on their street. The second asked whether the respondent believed the police on their block use more force than they have to. These items are also measured using a four-item Likert scale.

We employed one-tailed tests of statistical significance for these analyses as the treatment has a strong presumption of increasing PJ and legitimacy and reducing perceptions of harassment and use of police violence.

### Crime.

Crime incident data are ordinarily used by police for describing crime in communities but may underreport crime since police officers must write up a report that a crime has occurred. Citizen-initiated crime call data may overstate the extent of crime problems, since people may not always have accurate information about events they observe. Additionally, in programs that emphasize community engagement, there is some evidence that citizen reporting may be inflated relative to standard policing conditions (31). We assessed crime at the hot spots by examining both total crime incidents and total citizen-initiated crime calls (*SI Appendix*, section S1.10). Our measure for total crime incidents and total citizen-initiated crime calls included violent, property, drug, disorder, domestic, and other crimes (*SI Appendix*, section S1.10). We did not examine specific crime categories because the *N*s for analysis at the hot spot level become relatively small. We modeled crime outcomes using the standard log-link function for the negative binomial distribution because of overdispersion in crime counts in the intervention period. In the regression models estimated, *y* indicates the count of crime incidents or calls for service either during or after the intervention (depending on the model), *Group* is the treatment group the hot spot was assigned to, *City* is a dummy variable for city, *Block* is a dummy variable for statistical block used for hot spot randomization, and *Pretest* indicates the number of incidents or calls on the block in the six months preintervention:

$$(ln)y_{i}=B_{0}+B_{1}G\mathrm{roup}+B_{2-3}C\mathrm{ity}+B_{4-11}B\mathrm{lock}+B_{12}\text{log}\left(\mathrm{Pretest}\right)+e_{i}.$$

We compared the intervention period to a 6-mo preintervention period and the preintervention period to a 6-mo postintervention period. We employed two-tailed significance tests because there is not a strong presumption of the direction of treatment influence. While PJ advocates argue that procedurally just policing could reduce crime by enhancing legitimacy and increasing compliance with the law (9, 32), others argue that this softer version of policing could lead to crime increases (33, 34).

## Results

### Did the PJ Training Lead to More Procedurally Just Policing?

PJ group officers were significantly more likely to give people voice (*P* < 0.01), show neutrality (*P* < 0.05), and demonstrate respectful behavior (*P* < 0.05) in observed interactions. Cohen’s *d* values range between 0.22 and 0.39, suggesting standardized small to moderate treatment impacts (see Table 1; see the full model in *SI Appendix*, Table S24). Although our index of trustworthy motives fails to achieve statistical significance in these analyses, the impact is in the expected direction. The overall PJ index is strongly significant (*P* = 0.001), with the PJ group showing more procedurally just behavior (Cohen’s *d* = 0.39). Interactions involving SC group officers were significantly more likely to include disrespectful behavior (*P* = 0.01). In this case, Cohen’s *d* is moderate with a value of −0.51. Looking at PJ measures evidencing significant outcomes, we do not observe statistically significant variation across the three cities (*SI Appendix*, Table S25).

**Table 1.** Multilevel mixed-effects linear regression models for SSOs

Outcome (*n*)	PJ mean (SD)	SC mean (SD)	Adjusted mean difference	Cohen’s *d*	*P* value
Voice (327)	1.424 (0.700)	1.230 (0.780)	0.282	0.386	0.003
Neutrality (504)	0.943 (0.877)	0.895 (0.926)	0.196	0.219	0.016
Respect (503)	1.987 (2.319)	1.6 (2.120)	0.597	0.266	0.017
Trustworthy motives (502)	1.103 (1.185)	0.979 (1.064)	0.185	0.162	0.129
Overall PJ score (500)	27.761 (15.862)	23.801 (16.274)	6.180	0.386	0.001
Disrespect (503)	0.035 (0.216)	0.254 (1.010)	−0.325	−0.507	0.010

See *SI Appendix*, Table S24 for full model results. To calculate Cohen’s *d*, we ran ANOVAs that contained group (0 = SC, 1 = PJ) to obtain the pooled within-groups SD, which is the square root for the mean square (MS) of the residual on the ANOVA output. This number is the denominator. The numerator was the group coefficient in the model. Therefore, Cohen’s $d=\left(\text{group}\;\text{coefficient}/\sqrt{\text{residual}\;\text{MS}}\right)$. Group effect *P* values are based on one-tailed tests. Imputed values are used for voice in the overall PJ score (*SI Appendix*, section S1.7).

We report in *SI Appendix* on a series of sensitivity tests for these analyses. We analyzed data only for the interactions that occur within the project hot spots (*SI Appendix*, Tables S26 and S27). We also estimated the overall PJ index including only nonimputed values for voice (*SI Appendix*, section S1.7 and Table S28). These analyses produced very similar findings to those reported in Table 1.

### Did the Training Impact Arrest Behavior?

We also find that the PJ group officers were much less likely (*P* < 0.001) to make arrests during the experiment than the SC group officers (*SI Appendix*, Table S29). The IRR suggests a relative reduction in arrests of more than 60%. Arrest behavior does not vary significantly across the three cities (*SI Appendix*, Table S30), though few arrests were made in Cambridge (*SI Appendix*, section S2.4 and Table S19).

### Did the Training Impact Attitudes of People Living at the Hot Spots?

When comparing post- to preintervention surveys, the PJ and SC conditions differed little in regard to community perceptions of PJ or police legitimacy (on the block or citywide, see Table 2; for full models see *SI Appendix*, Table S31; for similar findings from an observational data study, see ref. 35). At the same time, people living in SC hot spots were significantly more likely to see police officers as harassing people on their block (*P* < 0.01) and using more force than necessary (*P* < 0.05). The effect sizes are small to moderate with a Cohen’s *d* value of −0.47 for harassment and −0.34 for police violence. We did not observe significant variability across the three cities for harassment or police violence (*SI Appendix*, Table S32). Analyses conducted using multilevel mixed-effects linear regression models (*SI Appendix*, Tables S33 and S34), or multilevel mixed-effects ordered logistic regression models (*SI Appendix*, Table S35) and partial proportional odds models (*SI Appendix*, Table S36) produced very similar results.

**Table 2.** Community survey findings for PJ, legitimacy, and police misbehavior

Outcome (*n*)	PJ post–pre mean (SD)	SC post–pre mean (SD)	Cohen’s *d*	*F* (num. df, denom. df)	*P* value
PJ and legitimacy					
PJ (117)	0.003 (0.276)	−0.022 (0.333)	0.097	0.28 (1,105)	0.299
Legitimacy on the block (118)	−0.015 (0.216)	0.012 (0.239)	−0.114	0.39 (1,106)	0.268
Legitimacy citywide (118)	0.015 (0.295)	−0.018 (0.291)	0.112	0.36 (1,106)	0.276
Police misbehavior					
Police harass or mistreat (116)	−0.058 (0.323)	0.077 (0.257)	−0.473	6.52 (1,104)	0.006
Police use too much force (117)	−0.114 (0.358)	0.009 (0.379)	−0.344	3.50 (1,105)	0.032

Means based on margins calculated in ANOVA models for each outcome. *n* represents the number of hot spots, *n* < 120 total when no postintervention survey data were available for a hot spot for a particular outcome. Cohen’s *d* calculated based on *d* = (*M*_2_ − *M*_1_) / *SD*_pooled_. One-tailed *P* value for F test for group effect. Full model is presented in *SI Appendix*, Table S31. df, degrees of freedom.

### Did the Training have Impacts on Crime?

We report in Table 3 a statistically significant 14% relative decline in total crime incidents in the PJ group hot spots as compared with the SC group hot spots during the experiment (*P* < 0.05; see full models in *SI Appendix*, Table S37). Given the increased number of arrests in the SC group (which would be associated with crime incidents), one explanation for this finding might be that it is due to increased enforcement behavior. However, in Cambridge, where few arrests were made overall, the observed treatment effect on crime incidents is larger than the average observed across the cities (*SI Appendix*, Table S38, though we did not find a significant treatment by city interaction, *SI Appendix*, Table S39). There was a 10% relative reduction in crime incidents observed in the PJ group hot spots comparing the pre- to postintervention period (see Table 4), but the effect was not statistically significant (full models in *SI Appendix*, Table S37). Analyzing the citizen-initiated call data, we do not observe significant outcomes. However, the observed effects are in the direction of relative crime reductions in the PJ hot spots (see Tables 3 and 4; full models in *SI Appendix*, Table S40).

**Table 3.** Negative binomial regression models for total crime incidents and total citizen-initiated crime calls comparing pre- and during intervention periods per hot spot

Crime type	PJ preintervention crime mean (SD)	SC preintervention crime mean (SD)	PJ during intervention crime mean (SD)	SC during intervention crime mean (SD)	IRR	*P* value
Total crime incidents	18.4 (18.362)	18.417 (26.004)	26.083 (28.807)	30.6 (43.774)	0.859	0.015
Total citizen-initiated crime calls	30.817 (30.341)	38.867 (53.971)	45.75 (46.034)	59.267 (91.083)	0.908	0.198

Total *n* = 120 hot spots. IRR calculated from the negative binomial regression models for each outcome. For full models, see *SI Appendix*, Table S36 (total crime incidents pre/during intervention) and *SI Appendix*, Table S39 (total citizen-initiated crime calls pre/during intervention).

**Table 4.** Negative binomial regression models for total crime incidents and total citizen-initiated crime calls comparing pre- and postintervention periods per hot spot

Crime type	PJ preintervention crime mean (SD)	SC preintervention crime mean (SD)	PJ postintervention crime mean (SD)	SC postintervention crime mean (SD)	IRR	*P* value
Total crime incidents	18.4 (18.362)	18.417 (26.004)	17.833 (20.377)	19.967 (29.864)	0.895	0.173
Total citizen-initiated crime calls	30.817 (30.341)	38.867 (53.971)	67.05 (91.653)	89.25 (165.911)	0.949	0.550

Total *n* = 120 hot spots. IRR calculated from the negative binomial regression models for each outcome. For full models, see *SI Appendix*, Table S36 (total crime incidents pre/post intervention) and *SI Appendix*, Table S39 (total citizen-initiated crime calls pre/post intervention).

Given that the COVID-19 pandemic was ongoing in Houston for part of the intervention period and the postintervention period, we also estimated our models excluding the Houston data for arrests, the community survey, and crime (*SI Appendix*, section S2.7; officer surveys and SSOs were collected before pandemic restrictions). Following our results described above that there were not significant treatment-by-city interactions for these analyses, the results excluding Houston showed the same general pattern as the full three city models (*SI Appendix*, Tables S41–S43).

## Conclusions

Can we improve police behavior at crime hot spots, where proactive policing interventions are carried out? The answer from our study is that we can through intensive police training in PJ. At the same time, such training is found to lead to fewer arrests, and to people reporting that the police are less violent and less harassing of people who live on their street. The fact that we also observe crime prevention benefits, at least in comparing the preintervention period with the intervention period is noteworthy, though that benefit is modest and only in regard to crime incident data.

The 2018 review of the National Academies of Sciences Committee on Proactive Policing (4) found that there was insufficient evidence to conclude that PJ policing will have impacts on crime or citizen attitudes. Our findings, from a multicity randomized trial conducted at places where interactions with police are most common, provide strong data for revising those conclusions. At the same time, we note that our findings do not support the overall process model of police legitimacy that has been key to the development of PJ policing (9–11). We do not find significant impacts of the intervention on police legitimacy measured either in terms of the hot spots themselves or the city overall. Another recent observational study of policing has produced similar outcomes regarding this relationship (35), suggesting that scholars need to reconsider issues of theory and measurement in understanding how procedurally just behavior impacts perceptions of legitimacy (36).

We also think that our study provides important insights regarding police training and its potential influence on police officer behavior. The National Research Council concluded in 2004 that “for many decades it has been assumed that more and better police training leads to improved police performance,” but that “few studies evaluate the impact of training programs on actual performance on the job” (37). More than a decade later, Skogan et al. reached a very similar conclusion, arguing “We know virtually nothing about the short- or long-term effects associated with police training of any type” (17). In reviewing the evidence base for the President’s Task Force on 21st Century Policing’s recommendations on training, Lum and colleagues concluded in 2016 that “additional research is needed in every area of training discussed in the Task Force recommendations. In most cases, we know little about the impact of these training programs on officer knowledge, attitudes, and behavior” (38). Our study provides strong experimental data confirming that police training can have important impacts on police behavior.

While we think our study strongly advances our knowledge about PJ policing, it is important to recognize that our findings are drawn from a trial in three specific cities, and in one city COVID-19 restrictions were in place for part of the treatment period and the collection of survey data. While we were able to successfully implement the study in three cities, given the diversity of America’s urban areas, replications of our experiment in other contexts are needed. We note as well the importance of studies in which all police service is provided by specially trained officers. One possible explanation, for example, for the lack of influence of treatment on perceptions of police legitimacy may be the continued presence on these streets of untrained patrol officers responding to emergency calls to the police. With these limitations in mind, our randomized trial points to the potential for PJ training not simply to encourage fair and respectful policing, but also to improve evaluations of the police and crime prevention effectiveness.

## Supplementary Material

Supplementary File

## Data Availability

Survey and SSO data, code, and materials for analyses presented in the main document and supplemental analyses are deposited on the Open Science Framework (https://osf.io/mh93z/files/) (39). All study data are available except official police data. For access to official police data, additional permissions are required through the individual police agencies.
