# Supplementary material for: Reforming the police through procedural justice training: A multicity randomized trial at crime hot spots
Source: Proc Natl Acad Sci U S A. 2022 Mar 28;119(14):e2118780119. doi: 10.1073/pnas.2118780119 (PMC9168920; doi:10.1073/pnas.2118780119)
Supplement: Supplementary File [file pnas.2118780119.sapp.pdf]

Supplementary Information for

Reforming the Police Through Procedural Justice Training: A Multi-City Randomized Trial at Crime Hot Spots.

David Weisburd[1,2]*, Cody W. Telep[3], Heather Vovak[4], Taryn Zastrow[1], Anthony A. Braga[5], Brandon Turchan[5]

[1]Department of Criminology, Law and Society, George Mason University; Fairfax, VA, USA.

[2]Institute of Criminology, The Hebrew University of Jerusalem; Jerusalem, Israel.

[3]School of Criminology and Criminal Justice, Arizona State University; Phoenix, AZ, USA.

[4]National Policing Institute; Arlington, VA, USA.

[5]Department of Criminology, University of Pennsylvania; Philadelphia, PA, USA.

*Corresponding author: David Weisburd

Email: dweisbur@gmu.edu

This PDF file includes:

S1: Methods
S2: Extended Description of Results
Figures S1 to S7
Tables S1 to S43

S1: Methods

We were able to implement the study successfully in three cities: Tucson, Arizona; Cambridge, Massachusetts, and Houston, Texas. We also began the intervention in Indianapolis, Indiana, but COVID-19 restrictions were ongoing from the start of implementation of the treatment. The intervention began on March 17, 2020, and after one week project officers were reassigned to patrol. The project re-started on June 27, 2020 and ended on November 6, 2020. Thus, the intervention was only implemented for about 4.5 months, or about one-half of the intended intervention period. Because of COVID-19 restrictions, we were also not able to collect systematic social observations even during the period when treatment was implemented. Below we report on methods used in the three sites where treatment was fully implemented and data collection was successfully carried out.

S1.1 Hot Spot Selection

We obtained two years of citywide crime incident data and citizen-initiated crime calls for service data for each city. Each dataset contained incidents or calls recorded at the address level along with XY coordinates. We categorized crime incidents and calls by six broad crime types: violent, property, drug, disorder, domestic, and other. Next, we geocoded and joined the crime data to the city's street centerline file using ArcGIS. Our match rate after address cleaning was 97% for Tucson, 96% for Cambridge, and 93% for Houston. We also obtained parcel data for each city to identify the number of residences on each street. Our inclusion criteria required at least 15 residences on the street. We screened segments using Google Maps imagery as a validation check.

We confirmed that street segments met the residency criterion through on-site visits to each segment. During these visits, researchers conducted a census of the street, counting the number of residences, and noting the presence of commercial land use, large apartment complexes, and general street activity. Researchers also documented all households on the street, noting any accessibility issues (such as locked gates or fences), and which households appeared to be vacant.

The criteria used as the threshold for crime at a hot spot was developed in consultation with police management in each city to allow for local differences in the way that crime hot spots were defined in each city (see below). We also met with police representatives in each city during the selection process to confirm that local police saw selected hot spots as appropriate for the intervention. While criteria for crime levels at hot spots were developed using crime incident data for violent, property, and drug incidents, after hot spot selection we examined citizen-initiated crime call information for violent, property, and drug calls to ensure that there was also a substantial number of citizen crime calls at all of the hot spots included in the study (see Tables S1-S6).

Tucson. We initially began with 23,713 streets in Tucson after geocoding and joining the crime data to the street centerline file. For a street to be included in Tucson, it must have experienced a minimum of five violent crime incidents in 2016; be ranked in the top 2% for violent, property, and drug crime incidents in the city overall in 2015 (25 crime incidents) and 2016 (28 crime incidents); experienced at least one crime in six of 12 months in 2015 and 2016; and experienced a minimum of 18 citizen-initiated crime calls for service on the street in 2016. These selection criteria resulted in 82 eligible segments. The Google Maps screening determined that 17 segments did not appear to contain 15 residential units and were excluded from the study. This resulted in 65 eligible segments.

Upon completion of the on-site census screening, an additional 15 segments were eliminated for the following reasons: the segment was more than 1,000 feet in length, or there were not 15 viable and accessible residential units. The remaining 50 segments were sorted using the number of 2016 violent, property, and drug crime incidents, and were selected in order of crime severity, while screening each eligible segment for contiguity to segments that had already been sampled, until the sample of 40 segments had been selected.

Cambridge. The Cambridge street centerline file contained 2,625 streets. To be included for eligibility in the study, Cambridge streets must have met the following criteria: at least 15 residences on each street; ranked in the top 15% for violent, property, and drug incidents in the city overall in 2016 (three crime incidents); experienced at least one crime in three of 12 months in 2016; ranked in the top 8% of violent, property, and drug crime incidents in 2017 (at least four incidents); experienced at least one crime in three of 12 months in 2017; and experienced a minimum of five citizen-initiated violent, property, or drug crime calls for service (top 8%). In addition, the segment had to be less than 1,000 feet long. These criteria resulted in 72 eligible segments.

Upon Google Maps validation and on-site censusing screening, 11 segments were eliminated, leaving 61 eligible segments. The 61 segments were sorted using the number of 2017 crime incidents and selected in order of crime severity while also screening each eligible segment for contiguity to segments that have already been sampled, until the sample of 40 segments had been selected.

Houston. We began with 67,076 streets in Houston. To be included for eligibility in the study, Houston streets must have met the following criteria: at least 15 residences on each street; ranked in the top 4% for violent, property, and drug incidents in the city overall in 2017 (at least five crime incidents); ranked in the top 2% of violent, property, and drug crime incidents in 2018 (at least 10 incidents); experienced at least one crime in six of 12 months in 2018; and experienced a minimum of eight citizen-initiated violent, property, or drug crime calls for service (top 5%). In addition, the segment had to be less than 1,000 feet long. These criteria resulted in 90 potentially eligible segments.

Once these initial segments were identified, a handful of streets in certain districts were excluded if they were north of Interstate 10, to avoid project officers needing to spend very large amounts of time traveling. Following the review in Google Maps and the census of the dwelling units, exactly 40 segments remained for inclusion in the study.

S1.2 Hot Spot Stratification, Statistical Power, and Randomization

Block randomization was used to increase the likelihood of probabilistic equivalence across the PJ and SC groups and to improve statistical power. The amount of crime a segment experienced was likely to influence key study outcomes: officer behavior, resident perceptions, and long-term crime reduction. While all segments identified in our study were crime hot spots, the 40 segments in each city experienced considerable variation in the amount of crime experienced. Using a blocked design guaranteed that each experimental condition would contain an equal number of segments that experienced comparable levels of crime. Once we identified our blocking strategy, we used a random number generator in Stata to create a random variable to assign random numbers to our segments (using Stata command *runiform()*). Then we sorted our segments by crime block and the random number to randomly assign half of the hot spots in each block to each group using the following equation in Stata:

*by crime_block: gen group = ceil(X * _n/_N)*, where X = the number of blocks.

We used CRT Power to assess the statistical power of the design. We developed the estimates assuming three cities (ICC=.30), four statistical blocks within each city (ICC=.50), and 120 hot spots overall. We used Tucson data to estimate interclass correlation coefficients because the data were available in the development stage of the study. Cohen's *d* effect sizes, with a .05 level of statistical significance were used for the calculations (with a .80 probability of observing a statistically significant outcome).

Tucson. We identified four statistical blocks in Tucson based on violent, property, and drug crime incidents from 2016: streets with 30-40 incidents (n=12), streets with 41-52 incidents (n=12), streets with 53-99 incidents (n=12), and streets with more than 100 incidents (n=4). Table S4 presents the four-block structure with the randomized final group assignment, as well as crime incident and citizen-initiated crime call totals for the same general crime categories.

Cambridge. We used a three-block structure in Cambridge for randomization, using violent, property, and drug crime counts from 2017: streets with four to five incidents (n=18), streets with six to eight incidents (n=12), and streets with nine or more incidents (n=10). Table S5 presents the three-block structure with the randomized final group assignment, as well as crime incident and citizen-initiated crime call totals for the same general crime categories.

Houston. We identified four statistical blocks in Houston based on violent, property, and drug crime incidents from 2018: streets with 10-13 incidents (n=16), streets with 14-20 incidents (n=12), streets with 23-38 incidents (n=6), and streets with 43-76 incidents (n=6). Table S6 presents the four-block structure with the randomized final group assignment, as well as crime incident and citizen-initiated crime call totals for the same general crime categories.

S1.3 Officer Selection and Allocation and Procedural Justice Training Protocol

In each city, the police department solicited volunteers for the project from patrol officers. Departments only told officers this would be a special nine-month project on reducing crime, and so officers had no advanced knowledge that the treatment in the project involved procedural justice training. We encouraged department leadership to choose officers who had at least a few years of experience, were unlikely to be promoted during the intervention, and had a reputation for being active on patrol, given that the project would primarily involve officers being proactive during noncommitted time. We did not ask departments to choose officers predisposed to community engagement or using procedural justice, though we recognize the potential for selection bias with volunteers and the ability of departments to screen these volunteers. After receiving a list of officers in each department (8 in Tucson and Houston, 12 in Cambridge), we randomly assigned officers to be in either the PJ group or SC group (see below). Thus, departments had no control over the treatment group assignment for participating officers.

In each city, we matched officers in pairs based on their background and experience characteristics and randomly assigned within these pairs. In Tucson, we matched based on pre-intervention assignment, because two officers worked in each of the department's four divisions before the project began. In Cambridge and Houston, we matched on gender (all officers in Tucson were male) and race/ethnicity. This matching procedure was designed to create balance in the groups in terms of officer background characteristics (e.g., gender and race/ethnicity) and experience (e.g., precinct or division worked in pre-intervention), though we recognize that ensuring balance is challenging with small officer sample sizes in each city. In Table S8 we show there are no large or statistically significant differences in background characteristics between the PJ and SC groups. We note that officer views about procedural justice are similar in the two groups based on officer survey responses (see Table S9).

In each city, we followed a similar approach in delivering the 40-hour procedural justice training on-site to the procedural justice (PJ) group officers. We began development of the training with a planning meeting held at the National Police Foundation (now known as the National Policing Institute) in November 2016. The meeting was attended by practitioner and academic experts on procedural justice training and included discussions about content to incorporate into the training, existing training models, and effective strategies for training delivery. The following experts participated in the meeting. Affiliations reflect organizational role as of November 2016:

- Dr. Theron Bowman, City Manager, City of Arlington, TX
- Superintendent Lisa Holmes, Chief of the Bureau of Professional Development, Boston Police Department
- Professor Stephen Mastrofski, Department of Criminology, Law and Society, George Mason University
- Sergeant Renée Mitchell, Sacramento Police Department
- Dr. Paul Quinton, Evidence and Evaluation Advisor, College of Policing (UK)
- Executive Director Sue Rahr, Washington State Criminal Justice Training Commission and former Sheriff, King County, WA

- Professor Dennis Rosenbaum, Department of Criminology, Law and Justice, University of Illinois at Chicago
- Professor Wesley Skogan, Department of Political Science, Northwestern University
- Professor Tom Tyler, Macklin Fleming Professor of Law and Professor of Psychology and Founding Director of The Justice Collaboratory, Yale Law School

The meeting also included representatives from the Tucson Police Department with expertise in training (Lt. Timothy Reese) and patrol operations (Lt. Thomas Hawke), who assisted in thinking through the logistics of starting the project in our initial city. Attendees contributed training materials that were used in other procedural justice projects in the U.S. and U.K. The final 40-hour curriculum drew upon these materials, while also incorporating additional material focused in particular on implementing procedural justice models in the context of hot spots policing. Published evaluations of these materials are cited when available.
The final training drew material from the following training protocols:

- Center for Court Innovation
- Center for Evidence-Based Crime Policy
- Chicago Police Department (1)
- Greater Manchester Police (College of Policing) (2)
- National Initiative for Building Community Trust & Justice (3)
- Police Executive Research Forum Integrating Communications, Assessment, and Tactics (ICAT) (4)
- Metropolitan Police Service (UK)
- Ohio Peace Officer Training Commission
- Office of Community Oriented Policing Services (University of Illinois)
- Quality Interaction Program (Chicago PD) (5)
- Sacramento Police Department
- Washington State Criminal Justice Training Commission Listen and Explain with Equity and Dignity (L.E.E.D.)

The project team delivered the training in each city. Training personnel from each participating agency reviewed the slides and gave recommendations, particularly on the module focused on trust in police in historical context. This session focused on trust from both a national and local perspective with customized examples based on high-profile events in the history of each agency. But the agencies were largely uninvolved in training delivery. The exact specifics for training varied somewhat by city. In Tucson, the training occurred over a two-week period (two ten-hour days each week), while in Cambridge and Houston, the training was delivered over one week (five eight-hour days). In Tucson and Cambridge, the training was delivered in a conference room, while in Houston it took place in a classroom at the police academy.

There were also varying levels of participation by supervisors. In Tucson, the sergeant working with the PJ group attended half of the training. In Houston, the sergeant attended all of the training. In Cambridge, a superintendent overseeing the PJ team attended most of the training. Officers received a binder with copies of all training slides and information sheets on their assigned hot spots (the same information on hot spots was given to the standard condition (SC) group as well) and were encouraged to refer to the binder throughout the project.

The training curriculum stayed largely the same across the three cities. The training was generally divided into approximately one-hour modules with breaks between each module. The modules included a mix of lecture from PowerPoint slides, video clips, and discussion of the slides and clips. Given the small group of officers, the training was very interactive with many opportunities for discussion about the videos and about how the training aligned with officer experiences. Sergeants and supervisors also played an important role in helping to reinforce training concepts. The main change between cities was that the lessons learned from Tucson were incorporated into training in Cambridge and Houston. These recommendations for changes to the curriculum were provided by Tucson project officers during an end of project de-brief. Additionally, some material was customized for each city, including the history module mentioned

above and specifics about the project (e.g., characteristics of the assigned hot spots and how activity logs would be completed).

The hour-by-hour training curriculum for Houston is shown in Figure S1. All materials can be accessed through Dropbox at:
https://www.dropbox.com/sh/y507kw0vdipmafp/AABsL6Lnnw4CwOhLva7oy4Wha?dl=0

S1.4 Officer Survey

We administered baseline surveys to all project officers on paper at the start of the training. Officers were assigned a two-digit project ID to use on this and subsequent surveys. Officers reviewed an informed consent statement before agreeing to participate. Surveys included questions about views of procedural justice, views of hot spots policing, communication style, and demographics. To avoid priming officers in the SC group about the PJ group training, we included only a small number of procedural justice questions on their survey at baseline.

At the end of the training, we surveyed PJ group officers with a very similar survey to identify whether the training in procedural justice influenced officer knowledge and attitudes. This allowed for pre- to post-training comparisons among PJ group officers. Given that the SC training was just four hours, we did not conduct an end of training survey with these officers.

S1.5 Officer Activity Logs

During the intervention in each city, project officers in both the PJ and SC groups completed a daily activity log to document their visits to the hot spot and project activities. Each officer completed a separate form for each shift, unless officers rode in the same police vehicle the entire shift, in which case they could collectively turn in one log. The logs were completed in a fillable PDF in Tucson and handwritten on a paper form in Cambridge. In Houston, a fillable PDF was used for the first half of the intervention, and the Houston Police Department created an internal database for log submission for the second half of the intervention. Logs were shared at least weekly with the group supervisor and the research team. Officers all worked the same shift times within cities, which included both afternoon and evening hours. In Cambridge, officers worked five 8-hour shifts per week. Because of the larger teams (six officers), there were at least two officers working seven days a week. In Tucson and Houston, officers worked four 10-hour shifts per week, and all project officers typically worked Wednesday-Saturday. We transferred log data to an Excel database for analysis.

Figure S2 is an example log from Tucson for the PJ group, which also included a reminder about the components of procedural justice at the top. The logs were viewed as a way to gather information about activities that may not appear in official data and included a description of officer activities. Officers were instructed to fill out a row of the log for each hot spot visited.

Based on the log data, we calculated the time spent in hot spots during visits. See Section S2.2 and S2.4 for an additional discussion of activity log analyses.

S1.6 Systematic Social Observation Methodology

What do procedurally just behaviors look like in the field? To answer this question, we conducted systematic social observations (SSOs) of police-citizen encounters. Following the SSO literature in policing, and our data collection instruments, we use the term citizen generically to denote any persons that have contact with the police. SSO entails a combination of qualitative and quantitative methods for observing and documenting police behavior. SSO offers an important method for assessing to what extent procedural justice training affected officer behavior and also a way to better understand police decision making in real time (6, 7). The strength of SSO versus other types of assessments of police behavior is that it offers an explicit method for observing a specific event and set of behaviors (7). In conducting SSOs, observers were trained to observe systematically using a protocol that provides clear definitions that allow for measurement and replication (6). We benefited in this part of our work from advice and guidance from Dr. Tal Jonathan-Zamir and Dr. Stephen Mastrofski, who are experts in this aspect of data collection, and were consultants in the SSO methodology and measures used for the study.

Data collected focused on actions taken by the officers while interacting with citizens during an encounter. Observers produced two types of data during these observations: (1) a

detailed narrative documenting and describing officer and citizen behavior, and (2) a detailed 93-item survey that quantified key aspects of the encounter and people involved.

Level of Observation and Measurement. In our study, observers documented and observed officer behaviors in encounters that occurred on ride-alongs with project officers during their shifts. The ride-along (Ride) comprised the entire observation session, normally lasting a full (eight to ten hour) work shift. We defined the encounter (Encounter) as a face-to-face interaction between the assigned officer and one or more members of the public (citizen) about police business that brought the two together in a significant interaction (in this study "citizen" is a generic term for a member of the public and is not intended to make any claim about an individual's citizenship status). The criteria for identifying encounters to be observed consisted of at least one of the following:

1) Lasted for at least one minute
2) Involved three verbal exchanges, OR
3) Involved the use of physical contact or threat of physical contact between officer and citizen.

Within each ride-along, the observers frequently witnessed more than one encounter between the officer and the public that met our criteria for coding. In the SSO narrative, the observers numbered the encounters sequentially and described each encounter in detail. Often, more than one citizen with whom a police officer had significant interaction was involved in an encounter. Observers numbered the citizens (Police/Citizen Interactions) sequentially in their narrative of the encounter (C1, C2, C3, etc.). Based on the written narrative, observers coded the quantitative coding instrument questions for every encounter, and every citizen/police officer interaction within that encounter. Each interaction with each citizen was coded as a police/citizen interaction. Table S17 shows the levels of observations used in the coding.

Types of Encounters. The types of encounters observed and documented by observers fell roughly into the following categories and mainly comprised those in which the officer engaged in:

1) Talking to business owners/apartment managers
2) Talking to residents informally in an effort to build rapport
3) Doing pedestrian/bicycle/traffic stops
4) Responding to a citizen-initiated dispatched call
5) Responding to a citizen flagging down the police for help
6) Initiating a casual encounter to communicate the project
7) Following up on leads/information received from citizens
8) Responding to a request for back-up

Observer Training. In each city, we hired and trained four to six individuals to conduct SSOs with the participating police department. Observers were primarily advanced undergraduate students, recent graduates, or graduate students. We provided observers with robust training to enable them to observe, document, and produce quality data in an ethical and safe manner. Training spanned multiple sessions and included online, in-person classroom, field, and webinar training. The training plan is provided in Figure S3.

Sample Selection and Field Procedures. SSO observers were assigned by research project management staff to ride along with one specific officer and tasked with paying close attention to how the officer interacted with the public. Even if the assigned officer had a partner or was working with several other officers, observers were tasked with keeping focus on their assigned officer. SSO observers were unaware to which condition officers were assigned.

In order to prevent selection biases by police managers or observers, we asked for schedules of officers a month in advance, and did not consult police managers in our allocation of observers to officers. In turn, the project managers assigning shifts did not consult with observers regarding which officers they would ride with, and they were blinded as to whether the officers

were in the PJ or SC group. To allow for time for officers to become familiar with their hot spots and for our observers to complete training, we did not start ride-alongs until month three of the intervention. We continued ride-alongs through the end of the intervention period in Tucson, throughout the intervention period in Cambridge with a concentration of rides in months three and four, and until in-person data collection had to be suspended due to COVID-19 in month five in Houston.

Because of the intensive training requirements of observers in SSO methods, there were only four-six observers active at any one time in each city. The small number of observers that were trained combined with the small number of officers in each group in each city, and our desire to balance the observations by officers, time, and observer, for the PJ and SC groups, meant that a simple randomization scheme for allocation of rides would not be possible. The scheduling challenges were compounded by the fact that observers needed at least two days off between observations to fully submit their narratives and quantitative codings.

At the start of each month of observation, project management staff completed a schedule for the city, matching up observers and officers, based on the availability of both. We rotated assignments between officers and observers to ensure that multiple observers observed each officer and that observers observed multiple officers. We also ensured that officers were observed at different points in the data collection period (i.e., in multiple months) and were observed on different days of the week. We also focused on ensuring our observers had similar numbers of rides with project officers from the PJ and SC groups. Officers in all three cities kept the same shift times during the project period, so there was no time of day variability within cities. All project officers provided informed consent to be observed.

Looking at the distribution of rides, we find that our allocation scheme created general equivalence between the groups. Our observers completed a total of 129 rides, 66 with PJ officers (51.16%) and 63 with the SC group (48.84%). We note that in Cambridge, several rides, particularly with SC officers, did not include any citizen interactions, and so our SSO analyses reflect data from 117 total rides (63 in the PJ group, 53 in the SC group). Each officer in the study was observed for at least two shifts. The average number of shift observations per officer was 4.607 (standard deviation [SD] = 1.133). There was not a significant difference in the average number of shifts observed by officer between the PJ and SC conditions (PJ mean = 4.500, SD = 1.286; SC mean = 4.714, SD = 0.994; $t = -0.493$, $p = .626$). There was also not a significant difference by group for the number of rides each day of the week officers were observed. In Tucson and Houston, officers worked Wednesday-Saturday shifts, so our observations were more likely to occur these days, with rides on all seven days in Cambridge. If we assign each day of the week a value 1 (Sunday) to 7 (Saturday), the mean value for day of the week is almost identical across groups (PJ mean = 5.000, SD= 1.437; SC mean = 5.061, SD = 1.323; $t= -0.249$, $p =.804$).

When we examined the number of rides per observer, there was variability in total rides, given that some observers did not work on the project the entire observation period. But among the 16 observers (6 in Tucson, 4 in Cambridge, 4 in Houston), 13 of them had complete balance between the groups in number of rides (i.e., had the same number of rides between PJ officers and SC officers, or had an odd number of total rides and so had one more ride with one of the groups). There was greater imbalance with three of the observers: two in Tucson (one who had 7 rides with the PJ group and 5 with the SC group and one who had 7 rides with the PJ group and 10 with the SC group), and one in Cambridge (who had 12 rides with the PJ group and 7 rides with the SC group). In these cases, we ran into logistical challenges in the field, for example, with some frequency we ran into situations in which rides had to be canceled last minute (due to changes in officer schedules, such as illness, court appearances, or other departmental training). To assess for any possible bias, we re-ran the multilevel mixed-effects linear regression model predicting overall procedural justice (see Table S24) three times, each time dropping the encounters recorded by one of these observers. Looking at our main outcome measure of procedurally just behavior, we do not see meaningful changes in the impact of group assignment. In Table S24 with all encounters included, we observe a group adjusted mean difference for overall procedural justice of 6.180 (standard error [SE] = 2.040) with $p < .01$). The effects are similar whether we drop the first (adjusted mean difference=6.078, SE = 2.308, $p < .01$) or

second (adjusted mean difference =6.592, SE =1.983, $p < .01$) observer in Tucson, or the observer in Cambridge (adjusted mean difference = 5.647, SE = 2.160, $p < .01$).

Data Collection and Quality. Observations consisted of three levels (ride, encounter, and citizen interaction) to be coded in two ways (qualitatively and quantitatively). At the end of each ride-along shift and based on the recollection of the police-community member interaction, observers were responsible for constructing a narrative of the event and responding to a set of structured questions about the interaction within 48 hours. Observers completed both the narrative and quantitative coding instrument for every ride.

Observers were provided guidelines for developing high quality narratives. These guidelines emphasized formatting; structured and consistent coding; chronological documentation; writing techniques for showing vs. telling; inclusion of important context (such as special circumstances); thick descriptions of the officer, environment, events, citizen, and interaction between the officer and the citizen; and key indicators of procedurally just or unjust behavior.

We asked each police department to give the observers as much access as possible to see and hear what happened between the assigned officer and the citizens they interacted with from beginning to end of encounter. Observers were instructed to follow the direction of their assigned officer about where to go and what to do during the shift, including staying in the car if requested by the officer. On occasion, this made it difficult for the observers to observe and document the police-citizen interaction. In these situations, observers were instructed to document to the extent possible. While observing police-citizen interactions, observers were instructed to take only enough notes to jog their memory about the encounter and to do so only outside of public view (e.g., when in the patrol car and not observing). Documentation was required to include time, location, and precipitating call information and was to be stripped of identifying names for participants in the encounter (e.g., police officer, citizen, bystander).

To ensure data quality, observers were encouraged to ask questions when unsure about how a certain measure should be coded or how to properly document an interaction. Each narrative was reviewed by a member of the project team. Reviews focused on the quality of the narrative and correspondence between the narrative and the quantitative coding of the encounter. Annotated narratives were returned to the observers with overall feedback and, if applicable, with specific items to address before the narrative was finalized.

Ethics of Data Collection. All observers completed the CITI human subjects research certification course for social-behavioral research (see https://about.citiprogram.org/en/course/human-subjects-research-2/). All officers provided their informed consent to be observed at the start of the ride-along. Observers were further trained on the requirements of ethical research and importance of adherence to the research protocols in place. Emphasizing the importance of acting as neutral and impartial observers, observers were prohibited from engaging in any of the following behaviors:

- Impersonating police, acting as a decoy, inviting an officer's attention to a crime or evidence of a crime (unless life or injury are being threatened), or solving the crime.
- Assisting beyond what a civilian would be required to do in extreme circumstances.
- Observers were not expected to be asked to operate a police vehicle or guard or watch citizens (unless in extremis situations) and if asked to do so, were instructed to decline.

Required Reporting. Observers who witnessed or observed child abuse were required to report this to the assigned officer and project team. Observers were also informed that in the course of their work on this project, there was the possibility of being required to testify in court as a witness. No instances occurred that fell under required reporting, nor did researchers receive court summons.

Communication and Safety. During the logistics training portion, observers were made aware of the importance of staying alert, situationally aware, present, and maintaining open lines of communication with their assigned officer and the project team at all times while in the field.

Observers were provided with examples of the types of situations they were likely to encounter and how to respond, including those benign, pleasant, undesirable, and/or potentially dangerous situations such as:

- Citizen curiosity about researcher presence, including questions about what the police are doing or what is going on.
- Harassment from citizens, bystanders, or even the police officers themselves and how they should respond in each case.
- Special circumstances such as personal illness, officer illness, special assignment (e.g., court), or an abbreviated ride.

S1.7 Systematic Social Observation Variable Construction

Our quantitative instrument for police-citizen interactions contained several questions for each of the four components of procedural justice (voice, neutrality, dignity and respect, and trustworthy motives). The measures were constructed as "formative indices" following earlier work by Jonathan-Zamir, Mastrofski, and Moyal (7), using SSO methods. Formative indices do not identify a single underlying structure, but rather bring together measures that reflect the construct of interest (8, 9).

We first created a composite index for each of the four procedural justice components as outlined below. Then, we used the four composite indices to create a scale for overall procedural justice demonstrated for each police-citizen interaction. Table S18 displays the means and standard deviations for each index by group.

Voice. We used two measures to create our overall index of giving citizens voice:

1) Did the officer ask the citizen to provide information or citizen's viewpoint of the event? (The question in the codebook read "Did the citizen provide information on his/her viewpoint to the police about the matter of interest in the encounter?" Because the officer could not control whether citizens provided their viewpoint, the responses we included in this measure reflect only the officer asking and the citizen's response to the officer). See below for a description of the imputed values used.
    - 0 = no, the officer did not request, and the citizen did not offer information/viewpoint
    - 1 = yes, the officer did request the citizen's viewpoint.

2) Was the officer an active listener? (The question in the codebook read: "How did the officer receive the citizen's information/viewpoint?" with the following answer options: dismissive listener, inattentive listener, passive listener, and active listener. To create the voice composite index, this question was recoded as a binary question on active listening, with dismissive, inattentive, and passive listening = 0, and active listener = 1).
    - 0 = No
    - 1 = Yes

The voice composite index summed measure one and measure two. The index range is from 0 (none) to 2 (high).

Neutrality. We used three measures to create our overall neutrality index:

1) Did the officer indicate that he/she would seek all viewpoints (first question) and not make a decision about what to do until he/she had gathered all necessary information (second question)? (This measure combines two questions from the coding instrument. First question: "What did the officer show to this citizen about his/her desire to hear all viewpoints about the matter at hand?" Second question: "Did the officer indicate to this citizen that he/she would not make a decision about what to do until he/she gathered all the necessary information?").

- 0 = The officer made no indication one way or the other ***OR*** the officer indicated he would exclude or discount this citizen's view ***OR*** the officer indicated that he would exclude or discount another citizen's view ***OR*** the officer indicated he would exclude or discount both this citizen's and another citizen's view (first question) ***OR*** No (second question)
- 1 = The officer indicated that he would seek all viewpoints (first question) ***OR*** Yes (second question)

2) Did the officer explain to the citizen why the police became involved in this situation?
    - 0 = No ***OR*** Yes, using justifications based on the officer's personal values about the people or events at hand
    - 1 = Yes, using justifications based on law and evidence, fact, assertations, public safety, the community's benefit, or the citizen's welfare ***OR*** Yes, a combination of justifications based on law and justifications based on the officer's personal values

3) Did the officer explain to the citizen the reasons behind police actions during the encounter or its resolution (that is, not just describing, but explaining/justifying the actions/resolution)?
    - 0 = No ***OR*** Yes, using justifications based on the officer's personal values about the people or events at hand
    - 1 = Yes, using justifications based on law and evidence, fact, assertations, public safety, the community's benefit, or the citizen's welfare ***OR*** Yes, a combination of justifications based on law and justifications based on the officer's personal values

The Neutrality composite index summed measures one through three. The index range is from 0 (no neutrality) to 3 (high neutrality).

Respect. We used two questions to create our overall respect index. For the first question with multiple answer options, it was coded as 0 if the behavior was not shown, and as 1 if the behavior was demonstrated. We then summed the four items. (The first question ranges from 0 – 4).

1) Did the officer show any respectful behavior to this citizen during the encounter? (Check all that apply)
    - Called citizen politely by name or title (Mr./Miss/Ma'am/Doctor)
    - Used polite requests/acknowledgements (Please, Thank you, May I…?, Would it be OK with you…?, I'd be grateful if you would…, I appreciate your…)
    - Identified self by name (e.g., "I'm Officer Reilly")
    - Other sign of respect (In 43 instances out of 203 one other sign of respect was shown, in two instances there were more than one other sign of respect demonstrated; e.g. shook hands with citizen, held the door open for citizens, told citizens to enjoy the weather)

The second question for respect was coded as follows:

2) What best characterizes the duration of the officer's respectful behavior to this citizen?
    - 0 = The officer showed no respectful behavior to the citizen
    - 1 = Brief: respectful acts occurred only once or twice
    - 2 = Intermittent: respectful acts occurred several times, but not continuously
    - 3 = Dominant: respectful acts occurred continuously throughout the encounter

We then took the summation from question one and multiplied it by question two. The Respect composite index ranges from 0 (no respectful behaviors) to 12 (high respectful behaviors).

Trustworthy Motives. We used one question to create this composite index. This was a multiple answer option question. Each behavior was coded as 0 if not shown, 1 if the behavior was shown.

1) Each of the following items relates to the degree of care and concern shown by the officer to this citizen during the encounter. Check each action that the officer took or promised to take for this citizen.
    - Asked about or showed concern for the citizen's well-being
    - Offered comfort of assurance to the citizen
    - Exerted control or influence over another person for the citizen
    - Filed a report for the citizen (exclude citations or other reports filed to document the citizen's arrest or his/her status as a suspect/wrongdoer)
    - Acted on behalf of the citizen with a government agency or private entity
    - Provided or arranged for physical assistance to the citizen (e.g., giving the citizen a ride to the detoxification center)
    - Provided advice on how the citizen could handle the situation or deal with the problem
    - Encouraged the citizen to seek additional assistance from the police for this or future problems
    - Promised to give the citizen's needs special attention in the future
    - Other display of care and concern not listed above (In 48 instances out of 502 the officer showed one other display of care and concern; e.g. spoke to citizen in native language, added the number for the police station to citizen's phone)

To create the Trustworthy composite index, we summed each of the ten items. The index ranges from 0 (no trustworthy motives demonstrated) to 10 (high trustworthy motives demonstrated).

Overall Procedural Justice Score. We developed our standardized overall procedural justice index by creating standardized scores for each of the composite indices described above. For each index, we divided the individual interaction score by the maximum index score, then multiplied by 100. This created a standardized score for each of our 4 procedural justice components.

1) Voice standardized score = the individual voice composite score divided by 2.37 (which reflects the maximum index score), then multiplied by 100. We used imputed values for missing voice values (see below). The stochastic mean replacement, as discussed below, adjusted our voice composite scale with a minimum index score of -.58, and a maximum index score of 2.37.
2) Neutrality standardized score = Neutrality composite index divided by three to account for the maximum index score, then multiplied by 100.
3) Respect standardized score = Respect composite index divided by 12 to account for the maximum index score, then multiplied by 100.
4) Trustworthy standardized score = Trustworthy composite index divided by ten to account for the maximum index score, then multiplied by 100.

The overall procedural justice score was then created by first summing the four standardized scores, then dividing that sum by four. The final score ranges from -.63 to 66.93 (because of the voice imputation we discuss below, some of the randomly imputed scores were a negative number).

Disrespect. We looked separately at disrespect. We used two questions to create our overall disrespect index. For the first question with multiple answer options, it was coded as 0 if the behavior was not shown, and as 1 if the behavior was demonstrated. We then summed the eight items. (The first question ranges from 0 – 8).

1) Did the officer show any disrespectful behavior to this citizen during the encounter? (Check all that apply)
   - Derogatory name calling, belittling remarks, slurs, cursing, obscene language
   - Unnecessarily loud voice
   - Interrupted citizen without apology
   - Impolite/obscene gestures, spitting
   - Sneering, eye-rolling, jokes at citizen's expense
   - Ignored citizen who sought attention, spoke curtly to citizen
   - Declined to identify self when citizen asked for name/badge number
   - Other signs of disrespectful behavior (In only eight cases out of 481 was one other sign of disrespect shown; e.g., heavy sighs, dismissive of citizen)

The second question for disrespect was coded as follows:

2) What best characterizes the duration of the officer's disrespectful behavior to this citizen?
   - The officer showed no disrespectful behavior to this citizen
   - Brief: disrespectful acts occurred only once or twice
   - Intermittent: disrespectful acts occurred several times
   - Dominant: disrespectful acts occurred constantly throughout the encounter

We then took the summation from question one and multiplied it by question two. While the disrespect composite index could range from 0 (no disrespectful behaviors) to 24 (high disrespectful behaviors), the maximum disrespect score in our sample was nine.

"Giving Voice" Imputation. Because of a relatively large number of missing values (n=181) for the measure of voice (due primarily to the fact that people did not necessarily actively engage or respond to officers, observers were unable to assess in these instances if officers were active listeners), we used an imputation method for allocation of voice values for the overall PJ scale. Scores for voice for other people in the same interaction are used when possible. For missing active listening values where there were other citizen-officer interactions in the encounter, we used that officer's non-missing active listening mean for that encounter. This method resulted in 52 missing value replacements, with 129 missing values remaining.

When mean encounter scores were not possible, the average scores for the officer across all interactions was used. We included a random error component in the imputations so that the standard deviation of the measure would not be reduced by the imputation approach. For the remaining 129 missing values, we calculated the mean non-missing active listening values for each encounter by officer. We then calculated the variance of the officer imputed active listening values for the 129 values by solving for the stochastic variance:

$$\text{Standard deviation of 129 values} = \sqrt{\text{variance imputed} + \text{variance stochastic.}}$$

Then, we subtracted stochastic variance from the variance of non-missing active listening values to obtain the stochastic distribution. We then generated a random number with a mean equal to zero and variance equal to the stochastic distribution for each interaction in Stata. After verifying that the mean was close to zero and the variance was close to the stochastic distribution, we added the random number to the imputed active listening value for the 129 missing values. Because the random number was generated by standard deviation, some of the values randomly generated were a negative number.

S1.8 Community Survey Methodology

In each of the cities, we conducted a survey of residents of the hot spots to assess public reaction to the intervention. We administered and completed 867 pre-intervention residential surveys and 637 post-intervention surveys in the three cities. We attempted to complete surveys at seven residences in each of the 120 project hot spots (40 in each city). Carrying out a census of the selected street segments (see below), we found that the total number of occupied

households on each street segment varied across the three cities from a median of 27 (Houston) to a median of 58 (Tucson). Accordingly, the sample on each street segment is a relatively large proportion of the population. When the sample is a large proportion of the population the standard errors of estimates gained are typically too large. We estimate that the finite population correlation varies between 88 and 94 percent of what it would be compared to the same size sample from a large or infinite population. We take a conservative approach and do not apply the finite population correction in part because the population sizes vary widely across street segments, and in part because of the smaller sample observed in Houston in the post-intervention period.

Residential Screening and Census. The first step in the community survey process involved taking a census to identify (1) dwelling units eligible to participate in the residential survey and (2) collecting enough information on each viable dwelling unit so that researchers would be able to go back to the dwelling to administer a residential survey provided the dwelling unit was sampled. The specific goals of the residential screening and census were to:

- Identify problems with the layout of the street segment that might make data collection difficult
- Identify dwellings that were vacant
- Identify occupied dwelling units so we could draw a random sample of households for the residential survey.

Training for Residential Census. Field researchers completed a two-day classroom and field training session that (1) introduced them to the methodology for conducting a residential census and (2) included field training in conducting a residential census. As part of the classroom training, field researchers were introduced to the differences between segments, buildings, addresses, and dwelling units.

As part of the field training, the researchers were tasked with:

- Finding an assigned segment
- Counting the residential addresses on that segment
- Coding problems with the segment
- Recording information about addresses not censused.

Data Quality and Validation. Because each city is unique, the identification of each street segment presented different complications in each city. Field researchers were encouraged to employ the following best practices for data collection:

1. Begin each residential census with a walkthrough of the segment to count number of addresses present and identify concerns.
2. Raise any questions about how to code a particular item, the layout of a street or any other issue with the project team.
3. Use of consistent coding and documentation practices.
4. Verification of residential dwelling unit count by field partner.

Each census conducted by the field researchers was validated by a member of the project team through a review of documentation and pictures. Figure S4 presents the residential census form.

Residential Survey. The purpose of the pre-intervention survey was to obtain a baseline measure of peoples' perceptions of the police. Based on the residential census, 15 households were randomly drawn from each eligible residential segment. The residential survey instrument comprised 200+ items nested in 60 question and took 20 to 30 minutes to complete per household.

Data collection procedures entailed:

1. Assessing the segment for safety upon arrival and checking in with the shift supervisor.
2. Following the list of assigned dwellings for each segment, including verifying the dwelling before knocking and attempting to make contact with the resident.
3. Following IRB-approved recruitment script to communicate the purpose of the study to the resident and obtain agreement to participate.
4. Obtaining informed consent (respondents read and signed a consent document) and confirming respondent eligibility (i.e., at least 18 years old and resided at the residence for at least three months) to take the survey for residents who agreed to take the survey.
5. Administering the survey on a tablet using the offline version of the Qualtrics survey application with the researcher reading all questions to the respondent and recording their responses on the tablet.
6. Providing the respondent with a $20 incentive payment.
7. Obtaining, when possible, contact information for the post-intervention follow-up survey.

If unable to make contact with a resident of the household, researchers documented the contact attempt and any important information to remember for subsequent contact attempts. At the end of the survey shift, researchers completed and submitted all survey documentation to include: uploading completed surveys, filing away completed contact sheets, logging remuneration payments, and adding appointments to the survey shift schedule. Figure S5 presents the residential contact form.

The post-intervention survey was fielded approximately nine to 12 months after the pre-intervention survey. As with the pre-intervention survey, we censused each hot spot to identify whether sample households were valid and accessible. As a result of the COVID-19 pandemic and social distancing requirements, data collection procedures in Houston were transitioned from in-person to telephone-based interviews.

In addition to conducting follow up surveys with pre-intervention survey respondents, sampling procedures for the post-intervention survey entailed randomly drawing additional households in each segment for survey participation. A different recruitment script was used for households that previously participated in the pre-intervention survey. In this case, researchers would ask for the pre-intervention respondent by first name (if the respondent had provided this during the pre-intervention survey) in an effort to survey as many of the same respondents in both waves.

Training for Residential Survey. Field researchers completed a two-day training session focused on (1) learning the methodology for conducting a community survey; (2) understanding the instrument questions, documentation procedures, and field logistics; and (3) running through multiple contact scenarios along with completing the documentation for each.

Field Logistics and Safety. Our field data collection required significant logistical preparation, teamwork, and oversight to ensure the collection of high-quality data in an ethical and safe manner. Every shift had a designated team leader responsible for overseeing procedures in the field and a project team shift manager on call. In order to ensure the safety of all while in the field, researchers were expected to adhere to the following protocol:

1. Work in teams of two to three people, never alone.
2. All data collection was to be conducted during daytime hours, never after dark.
3. Check in upon arrival to each segment (and departure), check in with the shift supervisor every 20 to 30 minutes and be reachable at all times.
4. Follow assigned segments for each shift and a pre-defined travel route, communicate any deviation from the pre-defined route.
5. Leave the hot spot immediately if feeling uncomfortable for any reason or unsafe conditions were encountered.
6. Follow explicit procedures for terminating an interview if they encountered risky, concerning, or dangerous situations inside the household and complete the survey outside whenever possible (though making sure that neighbors or others on the street could not listen in to the interview).

7. Carry a signed letter from the police department describing what researchers were doing in the area in case stopped by police officers.
8. Refer to the study's FAQ sheet to help answer questions regarding the study, why researchers were in the neighborhood or knocking on people's doors (Houston's FAQ sheet is shown in Figure S6).

COVID-19 Logistics. Due to the COVID-19 pandemic, data collection procedures were transitioned from in-person to telephone-based interviews for the post-intervention survey in Houston. Fieldwork in the post-intervention survey was limited to the residential census and cash remuneration drop offs. All field researchers were provided with personal protective equipment (PPE) to wear and use while in the field and were required to follow the CDC guidelines for maintaining six feet of social distancing.

Houston used the same residential survey as was used in the other cities, but we had to transition all surveying to telephone. As we noted in the main article, we utilized a combination of phone numbers provided by pre-intervention survey respondents and buying data from multiple companies to try to obtain phone numbers for respondents in each hot spot. We used the same procedures of assigning streets to our researchers and completing contact sheets for each household. Figure S7 is a sample contact sheet that has phone numbers associated with the address from either pre-intervention survey data or the research firms. Interviewers left voicemails and sent text messages with information on the survey if no potential respondent answered. In cases where the number was disconnected or a wrong number (went to a business), the number would be removed from the sample, but in cases of no answer or voicemail, the numbers would be called repeatedly throughout the survey period.

S1.9 Community Survey Outcome Variable Construction

We focus on five key outcome variables drawing from questions from the residential survey: (1) procedural justice on the block, (2) legitimacy on the block, (3) legitimacy citywide, (4) harassment and mistreatment, and (5) excessive use of force. This section presents the items that were included in each of these outcomes, as well as an explanation of how we dealt with missing data in constructing survey scales.

Survey Scale: Procedural Justice on the Block. The procedural justice on the block scale averaged responses from 12 statements all asked on question 31 of the resident survey, which asked respondents "Please tell me whether you strongly agree, agree, disagree, or strongly disagree with the following statements about the police on your block." The list of questions is included in Table S21. These items cover the four components of procedural justice emphasized in the officer training (voice, neutrality, dignity and respect, and trustworthy motives). Given the high correlations between items (Cronbach's α based on pre-intervention survey data = .92), we did not divide by procedural justice component and simply combined all 12 statements. For all items, response options were strongly agree (coded 4), agree, disagree, and strongly disagree (coded 1). Respondents could also say "don't know" or refuse to answer.

We averaged responses to these 12 questions for respondents who gave a response other than "don't know" for more than half (at least seven) of the 12 statements. In other words, respondents who skipped or said "don't know" to six or more of the 12 questions were dropped from the analysis. This led to dropping slightly more respondents from the SC group (12.6%) than the PJ group (10.2%) but the difference was not statistically significant ($p = .130$).

Survey Scale: Legitimacy on the Block. We combined six questions asked in question 32 of the resident survey to examine legitimacy perceptions on the block. In contrast to the citywide legitimacy scale described below, these questions focused on perceived legitimacy among officers working on the block the respondent lived on. Respondents were asked "Please tell me whether you strongly agree, agree, disagree, or strongly disagree with the following statements about the police on your block." We include the list of questions in Table S22. Because of low reliability when we separated questions more focused on obligation to obey from those focused on trust in police, we used a single combined scale (α at pre-intervention = .77). These questions were measured the same way as the procedural justice questions, and we followed the same

approach. We included those respondents who answered at least four of the six questions with a response other than "don't know." Missing data by group was almost identical (6.71% of cases in the SC group and 6.17% in the PJ group; p-value = .667).

Survey Scale: Legitimacy Citywide. For the citywide legitimacy scale, we combined responses from five statements asked on question 35 of the community survey (see Table S23). Here, respondents were asked "The next few questions are general questions about the CITY Police Department. Please tell me whether you strongly agree, agree, disagree, or strongly disagree with the following statements." Items were highly correlated (pre-intervention $\alpha = .92$). Questions were coded in the same way as the procedural justice questions, and we followed the same approach, including respondents who answered at least three of the five questions with a response other than "don't know." Missing data by group was almost identical (5.50% of cases in the SC group and 5.38% in the PJ group; p-value = .916).

Survey Single Questions. Two separate statements comprised the indicators for police harassment and police use of force: (1) "The police harass or mistreat people on my block," and (2) "Police officers on my block use more force than they have to."

For the two statements, we included respondents who answered with a response other than "don't know." Responses were coded in the same way as the other questions (strongly agree = 4 and strongly disagree = 1) though in this case, for both statements less agreement is indicative of more positive views of police. For the police harassment question, there were slightly more cases dropped because of missing values in the SC group (12.21%) than the PJ group (9.97%) though the difference was not statistically significant (p = .166). The same was true for the force statement (15.03% in SC group vs. 13.12% in PJ group). Again, this difference was not statistically significant (p = .286).

S1.10 Crime Incident and Citizen-Initiated Crime Call Outcomes

We obtained crime incident and citizen-initiated crime call data from each city that covered the intervention period, six months pre-intervention, and six months post-intervention. All data contained incidents or calls recorded to the address level along with XY coordinates. We categorized crime incidents and calls into six broad crime types: violent, property, drug, disorder, domestic, and other. We then geocoded and joined the crime and crime call data to the city's street centerline file using ArcGIS to identify the crime and crime calls that occurred on the project hot spots. After address cleaning, we obtained a match rate of 98% for Tucson, 93% for Cambridge, and 98% for Houston.

The total crime incident variable summed the six broad crime types: violent (20.82%), property (31.12%), drug (5.88%), disorder (24.11%), domestic (4.84%), and other (13.24%; e.g., harassment, threats). We compared the pre-intervention period to the intervention period, and to the post-intervention period. We summed the six broad crime types for the citizen-initiated crime call data into one total crime call variable: violent (17.19%), property (15.75%), drug (2.11%), disorder (28.48%), domestic (21.37%), and other (15.10%; e.g., harassment, threats). As with the incident data, we compared the pre-intervention period to the intervention period, and the post-intervention period.

S2: Extended Description of Results

S2.1 Officer Survey

As noted in S1.4, to avoid priming officers in the SC group about the PJ group training, we included only a small number of procedural justice questions on their survey at baseline. The baseline comparison between groups is presented in Table S9. The groups were very similar on these items before the training. At the end of the training period, we surveyed PJ group officers with a very similar survey. Though the sample size is small, the results suggest that the training influenced officer knowledge and views (Table S10). Using Wilcoxon matched-pairs signed rank tests 12 of the 17 questions showed statistical significance at the $p<.05$ level (one-tailed test).

Cohen's *d* values, calculated using paired t-tests, suggest generally moderate to large effects comparing pre- to post-training responses.

S2.2 Officer Activities

Officer Activity Logs. Based on the log data, we calculated the time project officers spent in hot spots during visits. We used these totals for our calculations of the proportion of time spent on the hot spots by our project officers (see main text discussion and below). We include summary data for each city by group in Table S11. We excluded any time spent on non-project activities (e.g., training, calls outside the hot spots that officers assisted with in an emergency situation). We also report both raw and adjusted total minutes—accounting for cases where officers rode together in a car. In Tucson, officers rode alone in a vehicle, and so these numbers are the same. But in Cambridge, officers almost always rode in pairs (or sometimes three to a car). In Houston, the PJ group officers typically rode in pairs. When officers rode in the same car, they typically submitted one log for the pair (or trio) and so, the raw minutes may underestimate the true number of minutes project officers spent in the hot spots.

Table S11 reports the number of adjusted minutes spent for each group at each city. Table S12 reports on a negative binomial regression model examining the effect of treatment group on adjusted minutes spent at the hot spots. We used the likelihood ratio test to determine that negative binomial models were more appropriate than Poisson models due to overdispersion. These likelihood ratio tests are presented below the negative binomial regression tables. These data showed strong evidence of overdispersion, suggesting negative binomial models were appropriate for modeling these count data. There is not a statistically significant difference in adjusted minutes spent by study condition for the study overall. To further explore differences by city, we included a negative binomial model with a group by city interaction for minutes spent in Table S13. A post-estimation Wald test does not show statistical significance for the interaction effect in the model.

Time Spent in Hot Spots. As would be expected assigning four or six officers to just 20 streets full-time, the PJ hot spots gained a substantial amount of police presence from project officers during the experiment (see Table S14). As noted in the main article, Houston police officers spent about half as much time in the hot spots as was the case in the other cities. We suspect that the much smaller number of minutes in Houston is related to the long distances that officers often had to travel between hot spots. We tried to minimize this somewhat by focusing only on hot spots in the southern half of Houston, but officers still often faced heavy traffic and long travel times in traveling from one hot spot to another. We measured presence in terms of individual officers, so that if two officers spent ten minutes together on the hot spots it would lead to a total of 20 minutes for this measure. Following our request during training that all hot spots receive a meaningful dosage of officer time during the intervention, all hot spots were treated during the nine-month experiment.

Time spent for the SC group is reported in Table S15. We note that the SC group officers were responsible for between 40% (Tucson) and 60% (Houston) of total policing in the SC condition hot spots. This is very similar to the results for the PJ group (see Table S14 and main text).

S2.3 Systematic Social Observation Full Models and Sensitivity Analyses

Project Hot Spot Analysis. In addition to the full models presented in the main article, we analyzed the police-citizen interactions that occurred only in project hot spots. The observers were unable to determine the location of 34 out of the 508 interactions, which resulted in a total of 474 interactions. Of those, 307 interactions occurred in the project hot spots. Table S16 presents the count of interactions by location and city. Tables S26 and S27 present the results for interactions that occurred only within the hot spots. The overall procedural justice measure is similar in magnitude to the main analysis (Cohen's *d*=0.42 versus 0.39). Neutrality is also very similar in both models (*d*=0.22 versus 0.20), as is voice (*d*=0.38 versus 0.39). In both models,

trustworthy motives is not significant (*d*=0.24 versus 0.16). Differences are somewhat larger for respect (*d*=0.17 versus 0.27).

Main Analysis. Our main study findings as reported in Table 1 are developed from the full SSO mixed models reported in Table S24. We also present the findings using the imputed and actual values for voice as described in S1.7 (see Table S28). As is apparent from the table, the results including or not including imputed values are very similar in magnitude for the main treatment group effects. To further explore differences by city we include multilevel mixed-effects models with a group by city interaction for the SSO main outcome variables that showed statistical significance in Table 1, shown in Table S25. Post-estimation Wald tests do not show significant differences for the city by group interaction effects in the models.

S2.4 Officer Arrests

Officers in the PJ group in all three cities made fewer arrests during the intervention than those in the SC group, though there were overall few arrests in Cambridge (see Table S19). We used the likelihood ratio test to determine that negative binomial models were more appropriate than Poisson models due to overdispersion. These likelihood ratio tests are presented below the negative binomial regression tables. Our negative binomial regression results in Table S29 show that PJ officers were significantly less likely to make arrests during the intervention. These results were similar when combining data from all three cities or when looking at just Tucson and Houston (given the small number of arrests overall in Cambridge). In these models, we controlled for the number of arrests made for officers in the six months leading up to the intervention to account for different officer propensities to make arrests. We used the natural log of pre-intervention arrests. Given that some Cambridge officers did not make any pre-intervention arrests, we added 0.5 to all pre-intervention arrest totals before logging.

In Table S30 we use a group by city interaction to explore differences in arrests by the three cities. A post-estimation Wald test suggests the interaction effect is not statistically significant.

S2.5 Community Survey Analyses

Table S31 includes the full ANOVA models that are used to produce Table 2 in the main article. Table S32 includes the ANOVA models with a test for group by city interaction for the police misbehavior outcomes that were statistically significant in Table 2.

In Tables S33 and S34 we conduct a sensitivity analysis for the ANOVA results. The models are multilevel mixed-effects linear regression models with weighting. These models include random effects for hot spot and household and fixed effects for time, group, a group by time interaction (the main parameter of interest for assessing treatment group effects), city, and block. We used probability weights that summed to one for the entire sample to better balance the relative contribution of each hot spot to the final model. Each hot spot was assigned an equal probability (1/120 = .0083) and each respondent within a hot spot was assigned a weight so that the number of respondents for each hot spot summed to .0083. Thus, in a situation where across pre-intervention and post-intervention, there were 14 respondents in a hot spot, each respondent received a weight of .000595.

In Table S35 we present additional sensitivity analyses using multilevel mixed-effects ordinal logistic regression models. Given that the two police behaviors rely on a single question measured on an ordinal Likert scale, we used these models as a sensitivity test, since our other mixed models assume linearity in the outcome variable. Brant tests and other tools for testing the proportional odds assumption in ordinal regression models are not usable in mixed models. We re-ran these models using partial proportional odds models in Table S36 to adjust for violations of the parallel lines assumption and results are similar, but those models did not allow for random effects or respondent weighting. These models included probability weights based on sample size of survey respondents for each hot spot.

S2.6 Crime Incident and Citizen-Initiated Crime Call Outcomes

Our main study findings, reported in Table 3 and Table 4 in the main text, were developed from the full negative binomial model for total crime incidents in Table S37. We used

the likelihood ratio test to determine that negative binomial models were more appropriate than Poisson models due to overdispersion. These likelihood ratio tests are presented below the negative binomial regression tables. We also ran negative binomial models to test for a group by city interaction, as presented in Table S39. A postestimation Wald test does not show statistical significance for the interaction effect in the crime incident model comparing the pre-intervention and intervention periods. We also developed a total citizen-initiated crime call variable that summed the six crime call types, as described in S1.10, again comparing the pre-intervention period to the intervention period, as well as the post-intervention period, reported in Table 3 and Table 4 in the main text. As shown in Table S40, we do not find significant changes based on group.

S2.7 Officer Arrest, Community Survey, and Crime Incident and Citizen-Initiated Crime Call Outcomes with Houston Omitted

Given the COVID-19 pandemic began having significant impacts in March 2020, during month five of the intervention period in Houston, and prevented in-person data collection for post-intervention resident surveys in Houston, we also analyzed outcomes omitting Houston data. Our purpose was to check whether the pattern of the findings held excluding Houston. We note that the statistical power of these findings is meaningfully reduced. COVID-19 did not impact systematic social observation data, because we conducted all ride-alongs in Houston by mid-March 2020. The main finding for arrest is that procedural justice group officers were much less likely to make arrests during the intervention. This pattern is also observed without the Houston sample. For the arrest analysis for the full study the IRR is .360 (see Table S29), and the sample without Houston is .448 (see Table S41). The main findings in the community survey were that residents of standard condition hot spots were more likely to perceive harassment and unnecessary use of force. This continues to be the case without Houston. Cohen’s *d* for harassment is -0.473 for the full study (see Table 2) and -0.479 for the sample without Houston (see Table S42). For excessive use of force the full study Cohen’s *d* was -0.344. and for the sample without Houston, it was -0.246. The main finding for the crime data is that crime incidents went down in the procedural justice hot spots relative to the standard condition hot spots comparing the pre and during period. For the crime incident outcome, the IRR for the full study for the comparison of the pre and during period is .859 (see Table 3), and for the sample without Houston is .903 (see Table S43).

Day 1: Monday, October 7, 2019

Overview of the project and the importance of trust in policing

Time (approximate):	***Topic:***
**8:00-9:00am**	Introduction to the hot spots project and officer survey
**9:10-10:10am**	Why hot spots policing?
**10:20-11:20am**	Why experimental outcome evaluations are important
**11:30am-12:30pm**	Lunch
**12:30-1:30pm**	Background on the project and plans for data collection
**1:40-2:40pm**	What is legitimacy? What is procedural justice? (part 1)
**2:50-3:50pm**	What is legitimacy? What is procedural justice? (part 2)
**3:50-4:00pm**	Wrap-up and close

Day 2: Tuesday, October 8, 2019

The importance of procedural justice and what do procedurally just citizen encounters with the police look like?

Time (approximate):	***Topic:***
**8:00-9:00am**	Trust in police in historical context
**9:10-10:10am**	Linking fair and effective policing
**10:20-11:20am**	Citizen voice/participation
**11:30am-12:30pm**	Lunch
**12:30-1:30pm**	Neutrality
**1:40pm-2:40pm**	Dignity and respect
**2:50-3:50pm**	Trustworthy motives
**3:50-4:00pm**	Wrap-up and close

Day 3: Wednesday, October 9, 2019

Practicing with procedural justice, the importance of verbal and nonverbal communication, and working with diverse populations

Time (approximate):	***Topic:***
**8:00-9:00am**	Pulling it together: what do procedurally just encounters with citizens look like?
**9:10-10:10am**	Applying procedural justice to scenarios
**10:20-11:20am**	Procedural justice role-play exercise
**11:30am-12:30pm**	Lunch
**12:30-1:30pm**	Looking at the viewpoints of others
**1:40pm-2:40pm**	Nonverbal communication
**2:50-3:50pm**	Working with individuals with behavioral health problems
**3:50-4:00pm**	Wrap-up and close

Day 4: Thursday, October 10, 2019

Working with diverse populations continued, and procedural justice and hot spots policing

Time (approximate):	*Topic:*
**8:00-9:00am**	Working with diverse populations
**9:10-10:10am**	Implicit bias and procedural justice: a refresher
**10:20-11:20am**	Perspectives on police from those who are incarcerated
**11:30am-12:30pm**	Lunch
**12:30-1:30pm**	Hot spots policing, crime, and perceptions of the police
**1:40pm-2:40pm**	Responding to crime hot spots with procedural justice (part 1)
**2:50-3:50pm**	Responding to crime hot spots with procedural justice (part 2)
**3:50-4:00pm**	Wrap-up and close

Day 5: Friday, October 11, 2019

Intervention planning and applying procedural justice in the field

Time (approximate):	*Topic:*
**8:00-9:00am**	Advice on project implementation from prior project sites
**9:10-10:10am**	Supervision plans
**10:20-11:20am**	Using the training checklist and activity log in the field
**11:30am-12:30pm**	Lunch
**12:30-1:30pm**	Practicing in the field (traveling to a project hot spot to practice using procedural justice in the field)
**1:30-2:30pm**	Practicing in the field (continued)
**2:40-3:40pm**	Wrap-up and final intervention planning
**3:40-4:00pm**	Officer survey and feedback

Figure S1: Example Procedural Justice Training Agenda

The hour-by-hour training curriculum from Houston is provided here as an example.

DAILY ACTIVITY LOG

<u>Procedural justice</u>: Voice/participation * Neutrality/transparency * Dignity and respect * Trustworthy motives/care and concern

Officer PR #: ________________ **Date: ______________________**

Shift Start Time: __________________Shift End Time: ______________________

Hot Spot Visits *Include all visits to assigned hot spots*

Start Time	End Time	Street Block (Segment ID)	Other Team Members (0 if none present)	911 Call Type (0 if not responding to call)	Description of Activities While in Hot Spot	Total # of Citizens Spoken to
			PR #s:	Type:		
			PR #s:	Type:		
			PR #s:	Type:		
			PR #s:	Type:		
			PR #s:	Type:		
			PR #s:	Type:		
			PR #s:	Type:		

Notes/Issues:

Page ____ of ______

Email log each day to supervisor and crime analyst

Figure S2: Example Daily Activity Log

This example activity log from Tucson from the PJ group includes a reminder about the components of the procedural justice at the top.

SSO Training Plan

Objectives:

The objectives of this training plan and schedule are to:

- Learn the methodology for conducting systematic social observation
- Become familiar with the study instruments and documentation protocols
- Practice conducting systematic social observation in the field
- Successfully obtain certification of training completion

Session 1: Classroom training (2 8-hour sessions)

Day 1: Led by Dr. Cody Telep

- Introduction to the concepts of procedural justice and police legitimacy
- Deep dive into procedural justice measures
- Ride logistics

Day 2: Led by Dr. Heather Vovak

- Admin/HR orientation
- Constructing systematic social observation narratives (what to do/not to do, notetaking and coding different levels of observation)
- Practice writing narratives

Session 2: Field training (completed over a 1-week period)

Day 1: Participate in one 4-hour practice ride-along with the Police Department

Day 2-3: Complete narrative write-up for practice ride (within 48 hours of ride-along)

Day 4: Heather Vovak and Maria Valdovinos provide feedback on narrative and refresher training as necessary

Day 5: Corrections are submitted and ride/observation is completed

Session 3: Webinar training (2 5-hour blocks)

Day 1: Introduction to the coding instrument, quantitative coding, using Qualtrics to enter data, practice coding (5-hour block, led by Dr. Heather Vovak)

Day 2: Additional practice coding narratives and problem codes, logistics refresher (5-hour block, led by Dr. Heather Vovak)

Session 4: Field training (completed over a 1-week period)

Day 1: Participate in one full 9-hour ride-along with the Police Department

Day 2-3: Complete narrative and quantitative data entry for full ride (within 48 hours of ride-along)

Day 4: Heather Vovak and Maria Valdovinos provides feedback on narrative

Day 5: Corrections are submitted and ride/observation is completed

Figure S3: Systematic Social Observation Training Plan

This example SSO training plan is from Houston.

Census Form

Segment ID: _______________
Segment address: ____
Cross Street 1: ____
Cross Street 2: ____

Researcher IDs: ______________ Date: _________________

Initial segment screening

1. Number of residential addresses not censused ___________
2. Number of non-residential addresses___________________
3. Number of residential addresses censused_______________

Segment problems (code with 0 or 1)

4. Not located in the right place_________________
5. Incorrect Cross streets(s)_____________________
6. Segment shape incorrect_____________________

7. Segment notes:
____

8. Addresses not censused

	A. Complex/ business name	B. Street address	C. Estimated # units	D. Contact information	E. Notes	F. Eligibility screen
1						
2						
3						

Eligibility screening codes

01: Student housing	06: Inaccessible, management on premise
02: Senior center	07: Inaccessible key fob
03: Residential treatment center	08: Inaccessible building lock
04: Locked fence or barrier cuts area off from observation	09: Public housing
05: Non-residential address	10: Other

Line	1. Address (including number and street)	2. # Dwell	3. Dwell desc.	4. Uninhab	5. Visit	6. Notes
0	123 Sample Ave	12	Apt a	0 or 1	English	Apartment a, building 1
0	123 Sample Ave (Could also be “”, or “same as above”)	12	Apt b	0 or 1	Spanish	Apartment b, building 1
1						
2						
3						
4						
5						
6						
7						
8						
9						
10						
11						
12						

Segment ID_______________

Cross Street 1 _________________________________

Cross Street 2_________________________________

Figure S4: Residential Census Form

This form was completed for every segment visited.

RECORD OF CONTACTS

HOUSEHOLD ADDRESS:	SEGMENT ID:
DESCRIPTION:	HOUSEHOLD ID:
CROSS STREET 1:	
CROSS STREET 2:	

SCAN DATE	INT. ID #	DATE	TIME	RESULT	NOTES
___/___	___ ___	___/___/___	___:____ AM/ PM	__ __	
___/___	___ ___	___/___/___	___:____ AM/ PM	__ __	
___/___	___ ___	___/___/___	___:____ AM/ PM	__ __	
___/___	___ ___	___/___/___	___:____ AM/ PM	__ __	
___/___	___ ___	___/___/___	___:____ AM/ PM	__ __	
___/___	___ ___	___/___/___	___:____ AM/ PM	__ __	
___/___	___ ___	___/___/___	___:____ AM/ PM	__ __	
___/___	___ ___	___/___/___	___:____ AM/ PM	__ __	
___/___	___ ___	___/___/___	___:____ AM/ PM	__ __	
___/___	___ ___	___/___/___	___:____ AM/ PM	__ __	
___/___	___ ___	___/___/___	___:____ AM/ PM	__ __	
___/___	___ ___	___/___/___	___:____ AM/ PM	__ __	
___/___	___ ___	___/___/___	___:____ AM/ PM	__ __	
___/___	___ ___	___/___/___	___:____ AM/ PM	__ __	
___/___	___ ___	___/___/___	___:____ AM/ PM	__ __	
___/___	___ ___	___/___/___	___:____ AM/ PM	__ __	
___/___	___ ___	___/___/___	___:____ AM/ PM	__ __	

INTERVIEW CODES (RESULTS)			
20	Come back later – made appointment	01	Completed interview
21	Come back later – no appointment	03	Incomplete interview – break off (final disposition; refused)
22	No answer and no contact	05	Physical or cognitive disability / impairment
23	Busy / initial refusal	07	Could not locate household
24	Incomplete interview – break off (revisit)	08	No way to get physical access to household
25	Language problem – translation needed (specify)	06	Specify:

4b Picture of unit sampled**

1. Respondent contact information:

Envelope ID: ______________________
Respondent name: ______________________
Phone number: ______________________
Email: ______________________

2. Accessibility problem? ☐

3. Dwelling assessment required? (Fill out items 4a and 4b) ☐

4a Provide a description of household location (if necessary):

____
____
____
____
____

**If no unit numbers are listed, units should be numbered from top to bottom and left to right

Vacancy Assessment

5. During ANY attempt to conduct a survey at this household, did a researcher notice any of the following indicators that the dwelling unit may be vacant?

Indicators	Indicator observed (check only once)
5a. A neighbor or someone who lives in the building told you that the dwelling unit was vacant.	☐
5b. Mail was overflowing from the mailbox or stacked on the ground, and it was old and sitting for a long time.	☐
5c. A realtor's combination lock was observed at the entrance of the property.	☐
5d. A casual look through the front window shows no furniture or personal belongings in the dwelling unit.	☐
5e. An eviction notice has been posted on the front door or window of the property.	☐
5f. It appears that doors or windows of the property have been recently boarded up.	☐
5g. Heavy construction or demolition work being done at the property suggests that the dwelling unit is not occupied.	☐
5h. The building has major structural problems and disrepair, such as a missing staircase, a caved-in roof or evidence of dumping.	☐
5i. A tree is growing through the foundation, roof or walls of this home or apartment building.	☐

Figure S5: Residential Contact Form

Each sampled household in the resident survey had a contact form. Every time the field research team visited that household to attempt a survey, the outcome of that visit was documented on this form.

RESIDENT VIEWS OF NEIGHBORHOOD SAFETY AND POLICE IN HOUSTON
FREQUENTLY ASKED QUESTIONS (FAQS)

1. How long will this take?	The survey takes about 20 minutes. We will give you $20 cash for your time. I can be quick.
2. How does this work?	If you are willing to participate, I will ask you a series of questions and record your responses on the tablet. Most of these are about your level of agreement with statements about your street block or about your interactions with and opinions about the police.
3. What's the purpose of this survey?	We're interviewing residents in Houston to learn more about resident views about neighborhood safety and the police.
4. Why did you pick me?	We selected a random sample of households across Houston. You were randomly selected to represent your community.
5. Who are you?	I'm a researcher working with Arizona State University and the National Police Foundation, which is a nonprofit research organization in Washington, DC.
6. Who is sponsoring this survey?	The study is funded by the Laura and John Arnold Foundation, a private foundation that helps fund criminal justice research projects. The study is not funded by government or taxpayer dollars.
7. How will this study help?	As a resident of Houston, you have an opportunity to voice your opinion

	about the conditions in your neighborhood. This study could affect programs in your local community.
8. What if I don't want to answer a question?	You don't have to answer any questions that make you feel uncomfortable, and you can stop at any time. Your answers are completely confidential.
9. Can I get a copy of the results?	Yes. If you are interested, we can email a copy of the results to you when the project is done. We will need your email address.
10. Do you work for the police?	No. We do *not* work for the Houston Police Department or any police agency; however, we will use the information you provide to help improve community-police relations in Houston.
11. Will this get anyone in trouble?	No. We do not want to know about any specific people and the answers you give are strictly confidential.
12. Who will know about this?	Eventually a report will be published, but no names, addresses, or locations will be used in the report.
13. Will you call me back again?	We are conducting the survey at 2 times, so we will reach out to you again at about this time next year for a follow-up survey.
14. Are you going to call my neighbors?	Only if they were randomly chosen.
15. Would you like to interview my spouse?	We only need to talk with the first available adult from each household. We'd be happy to talk with your

	spouse if you do not have time to talk with us.
16. How do I know this is legitimate?	The project director at Arizona State University is Dr. Cody Telep in the School of Criminology and Criminal Justice. He'd be happy to talk with you. His office phone number is 602.496.1295 or you can email him at cody.telep@asu.edu

Figure S6: Study Frequently Asked Questions Sheet for Residential Surveys

Field researchers were provided this sheet for the pre- and post-intervention surveys. This sheet contained common questions that researchers were asked about the study.

RECORD OF CONTACTS—VERSION 1

New contact	HOUSEHOLD ADDRESS:	SEGMENT ID:
	DESCRIPTION:	HOUSEHOLD ID:
	CROSS STREET 1:	PHONE NUMBER:
	CROSS STREET 2:	EMAIL:

CONTACT	INT. ID #	DATE	TIME	RESULT	NOTES
1	___	___/___/___	___:____ AM/ PM	__ __	
2	___	___/___/___	___:____ AM/ PM	__ __	
3	___	___/___/___	___:____ AM/ PM	__ __	
4	___	___/___/___	___:____ AM/ PM	__ __	
5	___	___/___/___	___:____ AM/ PM	__ __	
6	___	___/___/___	___:____ AM/ PM	__ __	
7	___	___/___/___	___:____ AM/ PM	__ __	
8	___	___/___/___	___:____ AM/ PM	__ __	
9	___	___/___/___	___:____ AM/ PM	__ __	
10	___	___/___/___	___:____ AM/ PM	__ __	
11	___	___/___/___	___:____ AM/ PM	__ __	
12	___	___/___/___	___:____ AM/ PM	__ __	
13	___	___/___/___	___:____ AM/ PM	__ __	
14	___	___/___/___	___:____ AM/ PM	__ __	
15	___	___/___/___	___:____ AM/ PM	__ __	
16	___	___/___/___	___:____ AM/ PM	__ __	
17	___	___/___/___	___:____ AM/ PM	__ __	

INTERVIEW CODES (RESULTS)			
20	Call back later – made appointment	01	Completed interview
21	Call back later – no appointment	09	Disconnected phone/wrong number
22	No answer – no voicemail	10	No answer – voicemail left
23	Busy / initial refusal	06	Other (Specify):
24	Incomplete interview – break off	25	Language problem – translation needed (specify)

1. Respondent contact information:
Respondent name: ______________________________
Phone number: ______________________________
Email: ______________________________

2. Remuneration delivery method (circle one):
Amazon gift card – email (email address should be provided above)
Amazon gift card – text message (phone number should be provided above)
Cash (verify address)

3. Remuneration delivery confirmation for Amazon gift cards (to be completed by shift manager upon delivery of gift card)
Gift card ID: ______________________
Date delivered:______________________
Delivered by:______________________

4. Remuneration delivery confirmation for cash (to be completed by shift manager upon delivery of cash)
Envelope ID:______________________
Date delivered:______________________
Delivered by:______________________
Access issues (describe):__________________

Figure S7: Post-Intervention Residential Contact Form

This contact form was used for the post-intervention Houston surveys, which were conducted by phone.

Table S1: Violent, Property, and Drug Incident and Citizen-Initiated Calls for Service Categories Used for Tucson Segment Eligibility

The crime categories listed here are a broad summary of crime categories from the Tucson incident and citizen-initiated call data. The raw crime data contained further detail, for example, aggravated assault was listed as aggravated assault by gun, aggravated assault by knife, and so on.

	Crime Incidents	
Violent	*Property*	*Drug*
Aggravated assault	Arson	Narcotic drug laws - possession
Assault	Burglary	Narcotic drug laws - sale
Homicide/Murder	Embezzlement	
Robbery	Forgery	
Sexual assault	Fraud	
	Car jacking	
	Larceny	
	Motor vehicle theft	
	Stolen property	

	Calls for Service	
Violent	*Property*	*Drug*
Aggravated assault	Burglary	Narcotics violation
Assault	Car jacking	Overdose
Car jacking	Embezzlement	
Fight	Fraud	
Robbery	Larceny	
Sexual assault	Motor vehicle theft	
Shooting		
Stabbing		
Weapons		

Table S2: Violent, Property, and Drug Incident and Citizen-Initiated Calls for Service Categories Used for Cambridge Segment Eligibility

The crime categories listed here are a broad summary of crime categories from the Cambridge incident and citizen-initiated call data. The raw crime data contained further detail, for example, aggravated assault was listed as aggravated assault by gun, aggravated assault by knife, and so on.

Crime Incidents		
Violent	*Property*	*Drug*
Aggravated assault	Arson	Drugs
Assault	Burglary	Overdose
Gun violation	Counterfeiting	
Homicide	Embezzlement	
Rape	Forgery	
Robbery	Larceny	
Weapons violation	Motor vehicle theft	
	Shoplifting	

Calls for Service		
Violent	*Property*	*Drug*
Assault	Breaking & entering	Drugs
Carjacking	Fare evasion	Overdose
Fight	Larceny	
Robbery	Motor vehicle theft	
Shooting	Shoplifting	
Stabbing		
Weapons violation		

Table S3: Violent, Property, and Drug Incident and Citizen-Initiated Calls for Service Categories Used for Houston Segment Eligibility

The crime categories listed here are a broad summary of crime categories from the Houston incident and citizen-initiated call data. The raw crime data contained further detail, for example, aggravated assault was listed as aggravated assault by gun, aggravated assault by knife, and so on.

	Crime Incidents	
Violent	*Property*	*Drug*
Aggravated assault	Arson	Equipment violation
Assault	Bribery	Narcotics violation
Homicide/Murder/Manslaughter	Burglary	
Robbery	Counterfeit/forgery	
Sexual assault	Fraud	
Weapons violation	Larceny	
	Motor vehicle theft	
	Pocket-picking/Purse-snatching	
	Shoplifting	
	Theft	
	Trespassing	

	Calls for Service	
Violent	*Property*	*Drug*
Assault	Arson	Disturbance - drugs
Robbery	Burglary	EMS - drugs
Sexual assault	Forgery/Fraud	
Shooting	Motor vehicle theft	
Suspicious person with weapon	Shoplifting	
Weapon disturbance	Theft	

Table S4: Tucson Four-Block Structure with Randomized Final Assignment

V/P/D represents the number of violent, property, and drug incidents and citizen-initiated calls. Block 1 = 12 segments with 30-40 incidents, Block 2 = 12 segments with 40-52 incidents, Block 3 = 12 segments with 54-79 incidents, and Block 4 = 4 segments with over 100 incidents. Group assignment: 1 = PJ group, 2 = SC group.

Street ID	*V/P/D Incidents 2016*	*V/P/D CFS 2016*	*Block Assignment*	*Group Assignment*
30255	183	56	4	1
8452	150	143	4	2
32598	120	45	4	1
22662	111	107	4	2
24204	79	67	3	2
37310	73	51	3	2
47314	68	73	3	2
27823	66	58	3	2
27257	61	65	3	2
23237	60	68	3	1
61339	60	51	3	1
5250	59	39	3	1
18686	58	55	3	1
51231	56	59	3	1
1900	55	48	3	1
2027	54	45	3	2
2943	52	36	2	1
21552	49	66	2	1
64834	47	41	2	2
26146	46	59	2	1
57186	45	48	2	2
8396	44	56	2	2
39731	43	43	2	1
34811	43	35	2	1
562	43	37	2	2
56593	42	33	2	1
21475	41	18	2	2
10949	41	42	2	2
7834	40	43	1	1
85258	39	34	1	1
20345	37	18	1	2
9783	36	43	1	1
13365	35	28	1	2
39186	34	30	1	2
24390	32	24	1	1
30639	32	41	1	2
7923	31	30	1	1
48413	31	20	1	1
9219	31	39	1	2
58957	30	25	1	2

Table S5: Cambridge Three-Block Structure with Randomized Final Assignment

V/P/D represents the number of violent, property, and drug incidents and citizen-initiated calls. Block 1 = 18 segments with 4-5 incidents, Block 2 = 12 segments with 6-8 incidents, and Block 3 = 10 segments with over 9 incidents. Group assignment: 1 = PJ group, 2 = SC group.

Street ID	*V/P/D Incidents 2017*	*V/P/D CFS 2017*	*Block Assignment*	*Group Assignment*
57	45	84	3	2
74	21	21	3	1
1482	14	23	3	1
444	13	15	3	2
748	12	33	3	1
1462	11	10	3	1
124	11	15	3	2
935	10	11	3	1
2324	9	19	3	2
67	9	9	3	2
771	8	8	2	2
1754	8	10	2	1
1004	8	11	2	1
1188	8	6	2	2
56	7	10	2	1
1186	7	12	2	2
1485	7	10	2	1
307	7	9	2	1
131	6	6	2	2
2295	6	8	2	2
2309	6	12	2	2
1748	6	5	2	1
158	5	6	1	1
241	5	8	1	2
393	5	7	1	1
758	5	9	1	1
1513	5	7	1	1
1582	5	9	1	2
2209	5	6	1	2
2253	5	5	1	2
2340	5	6	1	2
2402	5	15	1	1
802	4	8	1	1
1072	4	5	1	1
737	4	6	1	1
18	4	6	1	2
260	4	5	1	1
1753	4	6	1	2
894	4	6	1	2
2493	4	5	1	2

Table S6: Houston Four-Block Structure with Randomized Final Assignment

V/P/D represents the number of violent, property, and drug incidents and citizen-initiated calls. Block 1 = 16 segments with 10-13 incidents, Block 2 = 12 segments with 14-20 incidents, Block 3 = 6 segments with 23-38 incidents, and Block 4 = 6 segments with 43-76 incidents. Group assignment: 1 = PJ group, 2 = SC group.

Street ID	*V/P/D Incidents 2018*	*V/P/D CFS 2018*	*Block Assignment*	*Group Assignment*
85594	76	144	4	1
171634	59	43	4	2
113449	49	86	4	2
10107	46	80	4	1
137274	46	84	4	1
93357	43	77	4	2
187328	38	53	3	2
35113	28	45	3	2
99289	28	55	3	1
55887	27	28	3	1
31145	24	45	3	2
95413	23	42	3	1
191370	20	29	2	1
123126	18	30	2	2
44275	17	23	2	2
75814	16	25	2	2
9435	15	60	2	2
52266	15	20	2	1
83628	15	22	2	1
44793	15	42	2	2
1430	15	17	2	2
21174	14	29	2	1
36710	14	21	2	1
17538	14	26	2	1
95160	13	19	1	2
4825	13	30	1	2
72213	13	11	1	1
36907	12	15	1	1
52563	12	10	1	2
68009	12	26	1	2
121545	12	17	1	2
79800	11	30	1	2
36667	11	22	1	1
87157	11	15	1	1
82063	11	22	1	1
44592	10	14	1	2
44591	10	28	1	2
70097	10	19	1	1
87306	10	8	1	1
98213	10	17	1	1

Table S7: Community Survey Demographic Characteristics of Subjects at Crime Hot Spots by City

Demographics based on the pre-intervention survey.

Demographic	*Tucson Sample (n =329)*	*Cambridge Sample (n = 261)*	*Houston Sample (n = 277)*
Median age	41.0	34.0	43.5
% Female	55.6%	49.0%	58.8%
% Hispanic	36.7%	8.2%	26.3%
% Black	9.3%	11.7%	54.0%
% Asian	3.1%	13.6%	2.6%
% Married	16.5%	28.6%	29.2%
Median household size	2.0	2.0	2.0
% Bachelor’s or higher	17.6%	80.8%	33.2%
% Renting	87.2%	73.6%	53.8%
% Currently working	54.9%	69.6%	62.8%
% Household income ≤$25,000	68.6%	17.0%	42.5%

Table S8: Officer Characteristics in Procedural Justice and Standard Condition Groups (n=28)

P-value from two-tailed t-test for means and non-parametric median test for medians. The characteristics suggest no large or statistically significant differences by group at baseline.

Question	*PJ mean(SD)/ Proportion*	*SC mean(SD)/ Proportion*	*p-value*
Age mean	33.429 (10.256)	37.214 (12.192)	.382
Age median	31.000	31.000	.525
Years of experience mean	8.286 (10.276)	10.321 (11.572)	.627
Years of experience median	5.000	4.250	.890
Proportion female	.214	.143	.622
Proportion military experience	.357	.143	.204
Proportion bachelor’s degree	.643	.714	.699
Proportion Latino/Hispanic	.385	.214	.352
Proportion Black/African-American	.154	.286	.430
Proportion White	.538	.643	.598

Table S9: Officer Survey Baseline in Both Groups for Procedural Justice Questions (n=28)

Cohen's *d* is calculated from independent samples t-tests - $d = (M_2 - M_1) / SD_{pooled.}$ P-values are from two-sample t-tests.

Question	*PJ Mean (Std. dev.)*	*SC Mean (Std. dev.)*	*Cohen's d*	*p-value*
Officers shouldn't take time to listen to citizens complain about their problems	1.857 (.663)	1.929 (.997)	-0.085	.825
It is important to give everyone a good reason why we are stopping them, even if there is no legal requirement to do	3.643 (.497)	3.500 (.519)	0.281	.464
Officers should at all times treat people they encounter with dignity and respect	3.857 (.363)	3.857 (.363)	0	1.000
Police have enough trust in the public for them to work together effectively	2.714 (.611)	2.615 (.506)	0.172	.653

Table S10: Means, Cohen's *d*, and Wilcoxon Matched-Pairs Signed Rank Test for Pre- and Post-Training Responses by Procedural Justice Group Officers

Paired sample t-tests used for the calculation of Cohen's $d = t * \frac{\sqrt{2(1-r)}}{n}$ where *r* = correlation between pre and post test score). Wilcoxon matched-pairs signed rank test used for p-values. In all cases, the p-value for the t-tests and Wilcoxon tests were similar and the same 12 questions showed statistical significance at the p<.05 level (one-tailed test) in both sets of analyses.

Question (n)	*Pre-Mean (Std. dev.)*	*Post-Mean (Std. dev.)*	*Cohen's d*	*Negative Ranks (Sum)*	*Positive Ranks (Sum)*	*Ties (Sum)*	*p-value*
Voice							
Listening and talking to people is a good way to take charge of situations (14)	3.643(.497)	3.786(.426)	0.309	2(23)	4(46)	8(36)	.207
Letting people talk back only encourages them to get angrier (13)	2.615(.650)	2.077(.760)	-0.749	7(70)	0(0)	6(21)	.004
Officers need to show an honest interest in what people have to say, even if it is not going to change anything (14)	3.429(.514)	3.786(.426)	0.756	1(11)	6(66)	7(28)	.029
Officers shouldn't take time to listen to citizens complain about their problems (14)	1.857(.663)	1.500(.855)	-0.458	5(58)	1(11)	8(36)	.047
Neutrality							
It is important to give everyone a good reason why we are stopping them, even if there is no legal requirement to do (14)	3.643(.497)	3.929(.267)	0.683	0(0)	4(50)	10(55)	.023
If people ask why we are treating them as we are, we should stop and explain (14)	3.286(.469)	3.714(.469)	0.914	0(0)	6(69)	8(36)	.007
You can't always be open and honest when communicating with members of the public (14)	3.071(.616)	2.786(.699)	-0.431	5(57.5)	1(11.5)	8(36)	.051
When dealing with citizens' concerns, officers need to explain what will happen next, when they are done at the scene (14)	3.500(.519)	3.571(.646)	0.119	1(13)	2(26)	11(66)	.282
It is important that we remind people they have rights and that we appear to follow them (14)	3.357(.633)	3.857(.363)	0.948	0(0)	6(69)	8(36)	.007
It is very important that officers appear neutral in their application of legal rules (13)	3.615(.506)	3.923(.277)	0.722	0(0)	4(46)	9(45)	.023
Dignity and Respect							
Officers should treat citizens as if they can be trusted to do the right thing (14)	3.357(.633)	3.429(.646)	0.112	1(13)	2(26)	11(66)	.282
People should be treated with respect regardless of their attitude (13)	3.077(.760)	3.615(.506)	0.801	0(0)	5(55)	8(36)	.013

Officers should at all times treat people they encounter with dignity and respect (14)	3.857(.363)	3.857(.363)	0	1(13.5)	1(13.5)	12(78)	.500
Treating angry people with respect increases the community's confidence in the police (13)	3.077(.641)	3.692(.480)	1.075	0(0)	7(70)	6(21)	.004
In certain areas of the county, it's more useful for an officer to be aggressive than to be courteous (13)	2.154(.689)	1.615(.650)	-0.774	6(61)	1(9)	6(21)	.026
Trustworthy Motives							
Police have enough trust in the public for them to work together effectively (13)	2.692(.630)	2.923(.494)	0.393	0(0)	3(36)	10(55)	.042
There is no point in officers trying to convince some communities that the police can be trusted (14)	1.714(.469)	1.357(.497)	-0.738	5(60)	0(0)	9(45)	.013

Table S11: Minutes Spent in Hot Spots for PJ and SC Groups across Cities

Minutes raw include the calculation of minutes based on activity logs submitted. The minutes adjusted accounts for the number of officers per car. Minutes are multiplied when more than one officer rides in a car, but only one activity log was submitted for the shift.

	Tucson		***Cambridge***		***Houston***		***Total***	
	PJ	*SC*	*PJ*	*SC*	*PJ*	*SC*	*PJ*	*SC*
Minutes spent in hot spots (raw)	123,911	97,628	61,308	98,874	37,946	60,145	223,165	256,647
Minutes spent in hot spots (adjusted)	123,911	97,628	136,673	144,158	62,974	66,984	323,558	308,770

Table S12: Negative Binomial Regression Main Effects for Adjusted Minutes Spent by Hot Spot

Includes 120 hot spots. The IRR and standard error are reported for the adjusted minutes spent. The p-value reflects two-tailed tests: † $p < .10$. * $p < .05$. ** $p < .01$. *** $p < .001$. Below the table, the likelihood-ratio test that alpha equals zero, which compares this model to a Poisson model. The results strongly suggest that the alpha is non-zero and the negative binomial model is more appropriate than the Poisson model.

Variable	*IRR (Std. Error)*
Group	1.069 (.084)
Cambridge	.914 (.145)
Houston	.526 (.086)***
Block 2	.846 (.147)
Block 3	.826 (.144)
Block 4	1.456 (.359)
Block 6	1.233 (.196)
Block 7	2.000 (.336)***
Block 9	.768 (.125)
Block 10	1.148 (.234)
Block 11	1.730 (.353)**
Constant	5634.446 (736.031)
Ln(alpha)	-1.709 (.126)
Alpha	.181 (.023)

Likelihood-ratio test of alpha = 0: χ^2(df=1) =110,000, $p < .0001$

Table S13: Negative Binomial Regression Group by City Interaction for Adjusted Minutes Spent by Hot Spot

Includes 120 hot spots. The IRR and standard error are presented for the adjusted minutes spent. The p-value reflects two-tailed tests: † p < .10. * p < .05. ** p < .01. *** p < .001. The Wald Test of group by city significance χ^2 (df =2) = 4.67, p = .097. Below the table, the likelihood-ratio test that alpha equals zero, which compares this model to a Poisson model. The results strongly suggest that the alpha is non-zero and the negative binomial model is more appropriate than the Poisson model.

Variable	*IRR (Std. Error)*
Group	1.349 (.180)*
Cambridge	1.110 (.201)
Houston	.620 (.117)*
Block 2	.838 (.143)
Block 3	.825 (.141)
Block 4	1.535 (.373) †
Block 6	1.239 (.193)
Block 7	2.020 (.333)***
Block 9	.767 (.122) †
Block 10	1.141 (.229)
Block 11	1.710 (.344)**
Group x Cambridge	.681 (.128)*
Group x Houston	.734 (.138)
Constant	4973.248 (688.498)
Ln(alpha)	-1.746 (.126)
Alpha	.175 (.022)

Likelihood-ratio test of alpha = 0: χ^2(df=1) = 110,000, p < .0001

Table S14: Time Spent (in Minutes), and Percentage of Total Policing Provided by Procedural Justice Group

The official call data from Tucson did not include officer arrival to and departure from the scene times. However, we were able to obtain the number of minutes that the project officers spent on scene for six months prior to the intervention. We calculated the median for officer-initiated calls (18 minutes) and citizen-initiated calls (40 minutes). We then multiplied the number of the specific call type by its mean to obtain estimated times for the total calls for service minutes. To account for total minutes spent in Cambridge, we obtained official call data at the officer level. If two officers responded to a call, both officers were included in the count. We obtained the total calls for service minutes by subtracting the time the officer arrived at the scene from the time the officer cleared the scene. While we obtained the official call data in Houston, the data only listed times for one officer per scene. This resulted in us undercounting minutes for more serious calls where more than one officer would be on scene. We obtained the total call for service minutes by subtracting the time the officer arrived at the scene from the time the officer cleared the scene. Calls for service data included all citizen-initiated calls and all officer-initiated responses. Activity log minutes were calculated from the minutes in hot spots (adjusted) in the activity log data (see Table S11) and subtracting the CFS total minutes, so as not to double-count project officer activity on calls.

	Tucson		*Cambridge*		*Houston*	
	PJ Project officers	*Non-project officers*	*PJ Project officers*	*Non-project officers*	*PJ Project officers*	*Non-project officers*
Total CFS Minutes	16,488	131,576	4,635	133,621	4,264	39,881
Activity Log Minutes	107,423	0	132,038	0	58,710	0
Total Minutes	123,911	131,576	136,673	133,621	62,974	39,881
Percent of Total Policing Minutes	48.50%	51.50%	50.56%	49.44%	61.23%	38.77%

Table S15: Time Spent (in Minutes), and Percentage of Total Policing Provided by Standard Condition Group

The official call data from Tucson did not include officer arrival to and departure from the scene times. However, we were able to obtain the number of minutes that the project officers spent on scene for six months prior to the intervention. We calculated the median for officer-initiated calls (18 minutes) and citizen-initiated calls (40 minutes). We then multiplied the number of the specific call type by its mean to obtain estimated times for the total calls for service minutes. To account for total minutes spent in Cambridge, we obtained official call data at the officer level. If two officers responded to a call, both officers were included in the count. We obtained the total calls for service minutes by subtracting the time the officer arrived at the scene from the time the officer cleared the scene. While we obtained the official call data in Houston, the data only listed times for one officer per scene. This resulted in us undercounting minutes for more serious calls where more than one officer would be on scene. We obtained the total call for service minutes by subtracting the time the officer arrived at the scene from the time the officer cleared the scene. Calls for service data included all citizen-initiated calls and all officer-initiated responses. Activity log minutes were calculated from the minutes in hot spots (adjusted) in the activity log data (see Table S11) and subtracting the CFS total minutes, so as not to double-count project officer activity on calls.

	Tucson		*Cambridge*		*Houston*	
	SC Project officers	*Non-project officers*	*SC Project officers*	*Non-project officers*	*SC Project officers*	*Non-project officers*
Total CFS Minutes	52,876	143,812	5,441	123,930	2,537	45,577
Activity Log Minutes	44,752	0	138,717	0	64,447	0
Total Minutes	97,628	143,812	144,158	123,930	66,984	45,777
Percentage of Total Policing Minutes	40.44%	59.56%	53.77%	46.23%	59.40%	40.60%

Table S16: The Number of Interactions in Project Hot Spots versus Outside Project Hot Spots by City

Of the 474 interactions where observers could determine the location of the interaction, 304 occurred in a project hot spot.

Location	*Tucson*	*Cambridge*	*Houston*	*Total*
Outside project hot spots	99	8	60	167
Project hot spot	92	126	89	307
Total	191	134	149	474

Table S17: Systematic Social Observation Coding Levels

The three levels of observation codings were completed for each ride. Typically, each ride contained multiple encounters, and within those encounters, multiple citizens may have had interactions with the officer.

Level of observation and measurement	*Codes*
Ride (9 questions)	• Department/ride no./observer ID<br>• Date and start/end time of ride and observed shift<br>• Officer ID and assignment type
Encounter (23 questions)	• Location of encounter (e.g. address/property type/degree of observability)<br>• Observations and recordings (i.e. degree of observability and availability of body worn camera or other video recordings)<br>• Persons involved (e.g. interactional dynamics such as decision-making authority and supervisor presence; no. of people present including officers, citizens, and bystanders)<br>• Nature of problem (e.g. initiating factor, level of danger present etc.)
Police/Citizen Interaction (61 questions)	• Citizen characteristics (e.g. demographics and apparent socioeconomic status)<br>• Circumstances of encounter (i.e. role of citizen in encounter)<br>• State of citizen (e.g. citizen affect, behaviors, attitudes, actions, physical and emotional state etc.)<br>• Officer and citizen interactional dynamics (e.g. relationship, demands, responses, cooperation, use of force and presence of procedural justice indicators)

Table S18: Systematic Social Observation Procedural Justice Measure Descriptives by Group

Mean and standard deviation for each composite measure, along with the sample size for each measure. The overall PJ score included the imputed values for voice, as described in S1.7.

	PJ Group		***SC Group***	
Measure	*Mean (N)*	*Std. dev.*	*Mean (N)*	*Std. dev.*
Voice Composite	1.424 (205)	0.7	1.230 (122)	0.78
Neutrality Composite	.943 (314)	0.877	.895 (190)	0.926
Respect Composite	1.987 (313)	2.319	1.6 (190)	2.12
Trustworthy Composite	1.103 (312)	1.185	.979 (190)	1.064
Overall PJ Score	27.761 (311)	15.862	23.801 (189)	16.274
Disrespect	.035 (314)	0.216	.254 (189)	1.01

Table S19: Project Officer Arrests during the Intervention Period

The number of officers by city is listed in the city column. The number of arrests is reported by PJ and SC group. Arrest totals are based on official data provided by each police department. These data included arrests where a project officer was listed as the primary arresting officer.

City	*Procedural Justice*	*Standard Condition*
Tucson (n=8)	89	272
Cambridge (n=12)	4	5
Houston (n=8)	5	35
Three Cities (n=28)	98	312

Table S20: Pre-Intervention and Post-Intervention Survey Respondent Demographics in the Houston Community Survey

P-value based on two-tailed t-test for means and proportions and non-parametric median test for age median.

Demographic Factor	*Pre-Intervention (n=277) Mean (Std. dev.) / Proportion*	*Post-Intervention (n=108) Mean (Std. dev.) / Proportion*	*p-value*
Age mean	45.688 (16.407)	47.850 (16.593)	.250
Age median	43.5	48	.604
Household size mean	2.801 (1.542)	2.565 (1.262)	.157
Proportion female	.588	.620	.567
Proportion Latino/Hispanic	.263	.202	.221
Proportion Black/African-American	.540	.481	.303
Proportion White	.190	.264	.111
Proportion bachelor’s degree or higher	.332	.411	.147
Proportion own their home	.462	.596	.017

Table S21: Community Survey Procedural Justice on the Block Questions

Response items were strongly agree (coded 4), agree, disagree, and strongly disagree (coded 1). Respondents could also say “don’t know” or refuse to answer.

Question	*Procedural justice element*
Police officers explain their decisions to the people they deal with	Neutrality
The police are easy to talk to	Dignity/respect and Voice
Police officers don’t listen to all of the citizens involved before deciding what to do	Voice (reverse coded)
Police officers make decisions to handle problems fairly	Neutrality
The police provide opportunities for unfair decisions to be corrected	Neutrality
The police use rules and procedures that are fair to everyone	Neutrality
The police would treat you with respect if you had contact with them for any reason	Dignity/respect
Police on my block treat people with dignity and respect.	Dignity/respect
The police sincerely try to help people with their problems.	Trustworthy motives
Police on my block make decisions based on facts and the law and not on their own personal opinions.	Neutrality
Police officers address citizens in a respectful manner and an appropriate tone.	Dignity/respect
The police would help me if I called them.	Trustworthy motives

Table S22: Community Survey Legitimacy on the Block Questions

Response items were strongly agree (coded 4), agree, disagree, and strongly disagree (coded 1). Respondents could also say “don’t know” or refuse to answer.

Question
Most police officers who come to my block do their job well.
The police are generally honest.
You should accept police officers’ decisions even if you think they are wrong
The police are concerned with respecting citizens’ rights
The police should always be respected.
The police always have the right to make people obey the law.

Table S23: Community Survey Legitimacy Citywide Questions

Response items were strongly agree (coded 4), agree, disagree, and strongly disagree (coded 1). Respondents could also say “don’t know” or refuse to answer.

Question
I am proud of the CITY Police Department
I agree with many of the values that the CITY Police Department stands for
The work of the CITY Police Department encourages me to feel good about our city.
I have confidence in the police officers who patrol my city.
I trust the officers in the CITY Police Department

Table S24: Multilevel Mixed-Effects Linear Regression Models for Systematic Social Observations

The main study findings, reported in Table 1 in the main report, are developed from the full model presented here. The columns present the adjusted mean difference (standard error). Imputed values (see S1.7) are used for the voice score that was included in the Overall Procedural Justice score. One-tailed test for group effect only: † $p < .10$. * $p < .05$. ** $p < .01$. *** $p < .001$.

Variable	*Voice (n=327)*	*Neutrality (n=504)*	*Respect (n=503)*	*Trustworthy Motives (n=502)*	*Overall Procedural Justice score (n=500)*	*Disrespect (n=503)*
Group	.282 (.102)**	.196 (.091)*	.597 (.279)*	.185 (.163)	6.180 (2.038)**	-.325 (.140)**
Cambridge	-.224 (.123)†	-.925 (.111)***	-.872 (.339)*	-.179 (.196)	-11.463 (2.454)***	.194 (.167)
Houston	-.146 (.118)	-.344 (.104)**	-1.15 (.326)***	-.062 (.196)	-7.299 (2.414)**	.212 (.175)
Constant	1.28 (.093)	1.21(.083)	2.204 (.260)	1.028 (.158)	28.678 (1.929)	.208 (.140)
Random effects						
Officer	.012 (.019)	.002 (.013)	.112 (.126)	.081 (.044)	9.163 (7.227)	.094 (.038)
Encounter	.137 (.053)	.376 (.053)	3.49 (.380)	.451 (.093)	114.979 (17.410)	.236 (.038)

Table S25: Systematic Social Observation Multilevel Mixed-Effects Linear Regression Models with Group by City Interaction Terms

The columns present the adjusted mean difference (standard error). One-tailed test for group effect only: † $p < .10$. * $p < .05$. ** $p < .01$. *** $p < .001$. Trustworthy motives is not included in this analysis, as the group effect did not reach significance in the main analysis in Table S24.Wald Test of group by city significance for voice: χ^2 (df =2) = 1.23, p = .542. Wald Test of group by city significance for neutrality: χ^2 (df =2) = 4.80, p = .091. Wald Test of group by city significance for respect: χ^2 (df =2) = 1.47, .479. Imputed values are used for voice for the overall PJ score. Wald Test of group by city significance for overall PJ: χ^2 (df =2) = .35, p = .838. Wald Test of group by city significance for disrespect: χ^2 (df =2) = 2.24, p = .298.

Variable	*Voice (n=327)*	*Neutrality (n=504)*	*Respect (n=503)*	*Overall Procedural Justice score (n=500)*	*Disrespect (n=503)*
Group	.163 (.157)	.364 (.132)**	.655 (.419)†	5.935 (3.207)*	-.037 (.228)
Cambridge	-.288 (.199)	-.875 (.176)***	-1.108 (.523)*	-12.638 (3.756)**	.412 (.234)†
Houston	-.293 (.178)	-.081 (.158)	-.846 (.483)†	-6.604 (3.599)†	.449 (.239)†
Group x Cambridge	.130 (.253)	-.109 (.224)	.364 (.676)	1.965 (4.925)	-.413 (.318)
Group x Houston	.262 (.237)	-.443 (.205)*	-.494 (.639)	-1.066 (4.799)	-.458 (.330)
Constant	1.342 (.109)	1.124 (.095)	2.169 (.299)	28.784 (2.283)	.064 (.161)
Random effects					
Officer	.012 (.018)	8.52e-21 (.)	.087 (.119)	8.477 (7.147)	.081 (.034)
Encounter	.135 (.053)	.370 (.051)	3.491 (.380)	115.309 (17.445)	.236 (.038)

Table S26: Multilevel Mixed-Effects Linear Regression Models for Systematic Social Observations in Project Hot Spots Only

Cohen's *d* calculation is described in Table 1 in the main paper. The p-value is a one-tailed value from the mixed models. Imputed values are used for the voice score that was included in the overall PJ score.

Outcome (N)	*Procedural Justice Mean (Std. dev.)*	*Standard Cond Mean (Std. dev.)*	*Adjusted Mean Difference*	*Cohen's d*	*p-value*
Voice (195)	1.449 (.675)	1.206 (.764)	.266	0.377	.007
Neutrality (306)	.805 (.822)	.707 (.845)	.186	0.223	.058
Respect (306)	1.653 (2.191)	1.552 (1.810)	.345	0.168	.158
Trustworthy Motives (306)	1.068 (1.074)	.905 (.913)	.248	0.244	.057
Overall Procedural Justice score (306)	26.263 (13.953)	21.485 (14.920)	6.141	0.429	.002
Disrespect (306)	.032 (.228)	.207 (.704)	-.227	-0.483	.001

Table S27: Multilevel Mixed-Effects Linear Regression Models for Systematic Social Observations within Project Segments Only, Full Models

The full models from Table S26 are presented here. Imputed values (as described in S1.7) are used for the voice score that was included in the overall PJ score. One-tailed test for group effect only: † $p < .10$. * $p < .05$. ** $p < .01$. *** $p < .001$.

Variable	*Voice (n=195)*	*Neutrality (n=306)*	*Respect (n=306)*	*Trustworthy Motives (n=306)*	*Overall Procedural Justice score (n=306)*	*Disrespect (n=306)*
Group	.266 (.108)**	.186 (.118)†	.345 (.343)	.248 (.157)†	6.141 (2.057)**	-.227 (.074)**
Cambridge	-.173 (.120)	-.790 (.137)***	-.673 (.403)†	-.260 (.185)	-9.388 (2.401)***	.217 (.086)*
Houston	.040 (.136)	-.325 (.149)*	-1.381 (.439)**	-.490 (.202)*	-8.409 (2.610)**	.071 (.094)
Constant	1.254 (.102)	1.100 (.114)	2.098 (.340)	1.113 (.157)	27.411 (2.014)	.129 (.782)
Random effects						
Officer	8.48e-18 (7.39e-17)	.012 (.022)	.278 (.188)	.053 (.042)	4.862 (8.015)	.006 (.012)
Encounter	2.00e-15 (6.51e-51)	.362 (.065)	2.049 (.397)	.243 (.084)	76.210 (18.926)	.136 (.028)

Table S28: Systematic Social Observation Multilevel Mixed-Effects Linear Regression Models for Voice and Overall Procedural Justice, Original and Imputed Values

See S1.7 for the calculation of imputed values. The columns present the adjusted mean difference (standard error). One-tailed test for group effect only: † $p < .10$. * $p < .05$. ** $p < .01$. *** $p < .001$.

Variable	*Voice (original) (n=327)*	*Voice (encounter mean) (n=376)*	*Voice (imputed) (n=503)*	*Procedural Justice score (original) (n=325)*	*Procedural Justice score (encounter mean) (n=374)*	*Procedural Justice score (imputed) (n=500)*
Group	.282 (.102)**	.293 (.101)**	.283 (.101)**	6.135 (2.640)*	6.321 (2.718)*	6.180 (2.038)**
Cambridge	-.224 (.123)†	-.220 (.122)†	-.143 (.121)	-13.205 (3.164)***	-13.237 (3.261)***	-11.463 (2.454)***
Houston	-.146 (.118)	-.165 (.118)	-.191 (.121)	-5.958 (3.121)†	-6.523 (3.235)*	-7.299 (2.414)**
Constant	1.284 (.093)	1.238 (.093)	1.080 (.097)	35.323 (2.459)	34.419 (2.555)	28.678 (1.929)
Random effects						
Officer	.012 (.019)	.012 (.018)	.028 (.020)	15.061 (11.910)	17.801 (12.369)	9.164 (7.227)
Encounter	.137 (.053)	.214 (.047)	.195 (.039)	103.598 (25.193)	122.024 (23.443)	114.979 (17.410)

Table S29: Negative Binomial Regression Main Effect Results for Arrests by Officer during the Intervention Period

The Three Cities and Houston & Tucson only columns report the IRR (standard error). The p-value reflects two-tailed tests: † $p < .10$. * $p < .05$. ** $p < .01$. *** $p < .001$. Below the table, the likelihood-ratio test that alpha equals zero, which compares this model to a Poisson model. The results strongly suggest that the alpha is non-zero and the negative binomial model is more appropriate than the Poisson model.

Variable	*Three Cities (n=28)* *IRR (Std. Error)*	*Houston & Tucson only (n=16)* *IRR (Std. Error)*
Group	.360 (.093)***	.333 (.091)***
Pre-intervention arrests (Ln)	1.850 (.361)**	1.968 (.458)**
Cambridge	.131 (.094)**	-
Houston	.301 (.131)**	.329 (.159)*
Constant	5.837 (4.689)	4.778 (4.558)
Ln(alpha)	-1.799 (.541)	-1.867 (.529)
Alpha	.165 (.090)	.155 (.082)

Likelihood-ratio test of alpha = 0 for three cities: χ^2(df=1) =33.48, p < .0001
Likelihood-ratio test of alpha = 0 for Houston and Tucson only: χ^2(df=1) =32.76, p < .0001

Table S30: Negative Binomial Regression with Group by City Interaction for Arrests by Officer during the Intervention Period (n=28)

The IRR and standard error are reported. The p-value reflects two-tailed tests: † $p < .10$. * $p < .05$. ** $p < .01$. *** $p < .001$. The Wald Test of group by city significance χ^2 (df =2) = 2.84, p = .242. Below the table, the likelihood-ratio test that alpha equals zero, which compares this model to a Poisson model. The results strongly suggest that the alpha is non-zero and the negative binomial model is more appropriate than the Poisson model.

Variable	*IRR (Std. Error)*
Group	.397 (.121)**
Pre-intervention arrests (Ln)	1.821 (.350)**
Cambridge	.098 (.079)**
Houston	.368 (.172)*
Group x Cambridge	1.968 (1.546)
Group x Houston	.449 (.284)
Constant	5.923 (4.735)
Ln(alpha)	-1.911 (.541)
Alpha	.148 (.080)

Likelihood-ratio test of alpha = 0: χ^2(df=1) = 31.58, p < .0001

Table S31: Community Survey Full Main ANOVAs for Each Outcome

This table includes the full ANOVA models that are used to produce Table 2 in the main paper. The columns present the partial sums of squares (p-value). The p-value represents one-tailed test for group effect only.

Effect (df)	*Procedural justice-block (n=117)*	*Legitimacy-block (n=117)*	*Legitimacy-citywide (n=118)*	*Police harass/ mistreat (n=116)*	*Police use too much force (n=117)*
Model (11)	1.230 (.275)	.545 (.489)	.620 (.793)	1.446 (.122)	1.991 (.208)
City (2)	.279 (.220)	.011 (.903)	.086 (.618)	.028 (.849)	.132 (.612)
Block (8)	1.046 (.188)	.406 (.455)	.518 (.664)	.492 (.665)	.918 (.557)
Group (1)	.025 (.299)	.020 (.268)	.032 (.276)	.550 (.006)	.470 (.032)
Residual (104-106)	9.529	5.484	9.381	8.761	14.080

Table S32: ANOVAs for Police Misbehavior Outcomes with Group by City Interaction

The columns present the partial sums of squares (p-value). The p-values reported in the table below are non-directional.

Effect (df)	*Police harass/mistreat (n=116)*	*Police use too much force (n=117)*
Model (13)	1.566 (.162)	2.327 (.202)
City (2)	.429 (.085)	.624 (.102)
Block (8)	.468 (.700)	.897 (.570)
Group (1)	.550 (.006)	.470 (.032)
Group x City (2)	.120 (.495)	.336 (.288)
Residual (102-104)	8.641	13.744

Table S33: Multilevel Mixed-Effects Linear Regression Models for Procedural Justice and Legitimacy

The columns present the adjusted mean difference (standard error). One-tailed test for wave by group effect only: † $p < .10$. * $p < .05$. ** $p < .01$. *** $p < .001$.

Variable	*Procedural justice on the block (n=1,328)*	*Legitimacy on the block (n=1,328)*	*Legitimacy citywide (n=1,425)*
Wave	-.010 (.033)	.015 (.027)	-.004 (.030)
Group	-.021 (.037)	.018 (.029)	.006 (.035)
Wave x Group	.035 (.044)	-.019 (.038)	.037 (.044)
Block 2	.133 (.060)†	.092 (.047)†	.158 (.067)*
Block 3	.059 (.056)	.048 (.048)	.003 (.058)
Block 4	.093 (.059)	.047 (.062)	.077 (.055)
Block 6	.061 (.044)	.030 (.044)	.130 (.038)**
Block 7	-.128 (.050)*	-.087 (.050)†	-.106 (.049)*
Block 9	-.101 (.074)	-.039 (.061)	-.136 (.076)†
Block 10	-.116 (.106)	-.010 (.069)	-.111 (.095)
Block 11	.105 (.081)	.145 (.074)*	.055 (.088)
Cambridge	.161 (.050)**	.028 (.045)	.157 (.057)**
Houston	-.018 (.075)	-.036 (.055)	.032 (.077)
Constant	2.810 (.073)	2.750 (.055)	2.831 (.075)
Random effects			
Hot Spot	1.98e-22 (8.31e-21)	8.12e-20 (6.88e-18)	2.53e-19 (1.97e-17)
Household	7.94e-16 (6.82e-14)	1.37e-18 (1.16e-16)	9.25e-20 (7.53e-18)

Table S34: Multilevel Mixed-Effects Linear Regression Models for Police Misbehavior

The columns present the adjusted mean difference (standard error). One-tailed test for wave by group effect only: † $p < .10$. * $p < .05$. ** $p < .01$. *** $p < .001$.

Variable	*Police harass or mistreat (n=1,340)*	*Police use too much force (n=1,295)*
Wave	.047 (.033)	.010 (.038)
Group	.045 (.045)	.084 (.048)†
Wave x Group	-.108 (.051)*	-.114 (.060)*
Block 2	-.074 (.075)	-.125 (.074)†
Block 3	-.024 (.051)	-.014 (.075)
Block 4	-.107 (.077)†	.071 (.090)
Block 6	.055 (.052)	-.007 (.058)
Block 7	.185 (.068)**	.224 (.083)**
Block 9	.077 (.098)	-.005 (.083)
Block 10	.086 (.109)	-.048 (.117)
Block 11	-.183 (.097)†	-.192 (.126)
Cambridge	-.201 (.039)***	-.221 (.068)**
Houston	.071 (.068)	. 091 (.083)
Constant	1.939 (.060)	2.014 (.093)
Random effects		
Hot Spot	8.41e-17 (5.95e-15)	9.50e-21 (6.76e-19)
Household	5.86e-19 (4.67e-17)	6.14e-14 (4.33e-12)

Table S35: Multilevel Mixed-Effects Ordinal Logistic Regression Models for Police Misbehavior

The columns present the odds ratio (robust standard error). One-tailed test for wave x group effect only: † $p < .10$. * $p < .05$. ** $p < .01$. *** $p < .001$.

Variable	*Police harass or mistreat (n=1,340)*	*Police use too much force (n=1,295)*
Wave	1.333 (.218)†	1.071 (.184)
Group	1.207 (.241)	1.444 (.322)†
Wave x Group	.615 (.148)*	.606 (.165)*
Block 2	.639 (.223)	.512 (.191)†
Block 3	.897 (.246)	.853 (.313)
Block 4	.616 (.236)	1.289 (.580)
Block 6	1.285 (.322)	.950 (.261)
Block 7	2.310 (.766)*	2.616 (1.061)*
Block 9	1.511 (.661)	1.026 (.406)
Block 10	1.573 (.752)	.746 (.372)
Block 11	.435 (.204)†	.395 (.273)†
Cambridge	.398 (.079)***	.343 (.117)**
Houston	1.389 (.452)	1.548 (.627)
Cut 1	-1.707 (.298)	-2.200 (.460)
Cut 2	2.580 (.323)	2.191 (.451)
Cut 3	4.910 (.417)	4.243 (.502)
Random effects		
Hot Spot	4.70e-36 (1.12e-35)	2.24e-35 (4.37e-35)
Household	4.92e-36 (1.44e-35)	1.88e-35 (1.65e-34)

Table S36: Partial Proportional Odds Models for Police Misbehavior

The columns present the coefficient (robust standard error) Reference group is strongly agree. Models are generalized ordered logit/partial proportional odds models (10). One-tailed test for wave x group effect only: † $p < .10$. * $p < .05$. ** $p < .01$. *** $p < .001$.

Variable	*Police harass or mistreat (n=1,340)*	*Police use too much force (n=1,295)*
Strongly disagree		
Wave	.460 (.228)*	.064 (.197)
Group	.183 (.173)	.359 (.187)†
Wave x Group	-.503 (.280)*	-.495 (.281)*
Block 2	-.421 (.262)	-.670 (.280)†
Block 3	-.103 (.263)	-.159 (.279)
Block 4	-.459 (.312)	.254 (.381)
Block 6	.303 (.297)	-.053 (.293)
Block 7	.599 (.341)†	.821 (.385)*
Block 9	.382 (.312)	.025 (.320)
Block 10	.413 (.339)	-.295 (.378)
Block 11	-.593 (.470)	-1.266 (.464)**
Cambridge	-.733 (.262)**	-1.010 (.292)**
Houston	.292 (.278)	.438 (.317)
Constant	1.391(.385)	2.221 (.385)
Disagree		
Wave	.088 (.253)	.064 (.197)
Group	.183 (.173)	.359 (.187)†
Wave x Group	-.503 (.280)*	-.495 (.281)**
Block 2	-.421 (.262)	-.670 (.280)†
Block 3	-.103 (.263)	-.159 (.279)
Block 4	-.459 (.312)	.254 (.381)
Block 6	.303 (.297)	-.053 (.293)
Block 7	1.807 (.518)***	1.372 (.491)**
Block 9	.382 (.312)	.025 (.320)
Block 10	.413 (.339)	-.295 (.378)
Block 11	-1.173 (.638)†	-.425 (.449)
Cambridge	-1.807 (.445)***	-1.437 (.411)***
Houston	.292 (.278)	.438 (.317)
Constant	-2.207 (.433)	-2.182 (.399)
Agree		
Wave	-12.656 (.317)***	.064 (.197)
Group	.183 (.173)	.359 (.187)†
Wave x Group	-.503 (.280)*	-.495 (.281)*
Block 2	-.421 (.262)	-.670 (.280)†
Block 3	-.103 (.263)	-.159 (.279)
Block 4	-.459 (.312)	.254 (.381)
Block 6	.303 (.297)	-.053 (.293)
Block 7	13.824 (1.033)	13.805 (.609)

Block 9	.382 (.312)	.025 (.320)
Block 10	.413 (.339)	-.295 (.378)
Block 11	-13.289 (.561)***	-12.091 (.347)***
Cambridge	-13.835 (.466)***	-12.991 (.342)***
Houston	.292 (.278)	.438 (.317)
Constant	8.661 (.618)	-4.21(.455)

Table S37: Negative Binomial Regression Models for Total Crime Incidents

This table presents the full negative binomial regression models that are shown in Table 3 and Table 4 in the main paper. 120 hot spots included in the analysis. The columns present the IRR (standard error). P-values are two-tailed tests: † p < .10. * p < .05. ** p < .01. *** p < .001. The pre-intervention crime counts were logged values. Counts of 0 were changed to 0.5, for a logged value of -0.69. Below the table, the likelihood-ratio test that alpha equals zero, which compares this model to a Poisson model. The results strongly suggest that the alpha is non-zero and the negative binomial model is more appropriate than the Poisson model.

Variable	*Total Crime pre/during intervention*	*Total Crime pre/post intervention*
Pre-Intervention Crime	2.075 (.106)***	1.941 (.121)***
Group	.858 (.054)*	.895 (.073)
Tucson	1.184 (.213)	1.724 (.403)*
Cambridge	.858 (.143)	.897 (.198)
Block 2	1.211 (.134)†	1.120 (.161)
Block 3	1.204 (.132)†	1.239 (.176)
Block 4	1.344 (.209)†	1.403 (.284)†
Block 6	.967 (.167)	.978 (.221)
Block 7	1.217 (.217)	1.262 (.291)
Block 9	.499 (.094)***	.726 (.172)
Block 10	.858 (.168)	1.013 (.264)
Block 11	1.181 (.237)	2.118 (.529)**
Constant	3.315 (.437)	1.945 (.339)
/lnalpha	-2.973 (.282)	-2.396 (.276)
Alpha	.051 (.014)	.091 (.025)

Likelihood-ratio test of alpha = 0 for pre/during intervention model: $\chi^2(df=1) = 59.22$, $p < .0001$
Likelihood-ratio test of alpha = 0 for pre/post intervention model: $\chi^2(df=1) = 78.13$, $p < .0001$

Table S38: Cambridge Negative Binomial Regression Model for Total Crime Incidents

This table shows the analysis comparing pre-intervention crime incidents for Cambridge to total crime incidents during the intervention. N=40 hot spots. The column presents the IRR (standard error). P-values are two-tailed tests: † p < .10. * p < .05. ** p < .01. *** p < .001. The pre-intervention crime counts were logged values. Counts of 0 were changed to 0.5, for a logged value of -0.69. Below the table, the likelihood-ratio test that alpha equals zero, which compares this model to a Poisson model. The results strongly suggest that the alpha is non-zero and the negative binomial model is more appropriate than the Poisson model.

Variable	*Total Crime pre/during intervention*
Pre-Intervention Crime	1.738 (.178)***
Group	.787 (.113)†
Block 6	1.077 (.211)
Block 7	1.630 (.410)†
Constant	3.607 (.626)
/lnalpha	-2.462 (.472)
Alpha	.085 (.040)

Likelihood-ratio test of alpha = 0: χ^2(df=1) = 16.56, p < .0001

Table S39: Negative Binomial Regression Models for Total Crime Incidents with Group by City Interactions

120 hot spots included in the analysis. The columns present the IRR (standard error). P-values are two-tailed tests: † p < .10. * p < .05. ** p < .01. *** p < .001. Wald test of group by city significance for pre/during intervention: χ^2 (df =2) = 4.65, p = .098. Wald test of group by city significance for pre/post intervention: χ^2 (df =2) = 2.03, p = .363. The pre-intervention crime counts were logged values. Counts of 0 were changed to 0.5, for a logged value of -0.69. Below the table, the likelihood-ratio test that alpha equals zero, which compares this model to a Poisson model. The results strongly suggest that the alpha is non-zero and the negative binomial model is more appropriate than the Poisson model.

Variable	*Total Crime pre/during intervention*	*Total Crime pre/post intervention*
Pre-Intervention Crime	2.095 (.106)***	1.937 (.121)***
Group	.711 (.096)*	1.037 (.180)
Tucson	1.017 (.194)	1.830 (.467)*
Cambridge	.853 (.158)	1.073 (.271)
Group x Tucson	1.352 (.213)†	.880 (.180)
Group x Cambridge	1.083 (.201)	.711 (.173)
Block 2	1.213 (.130)†	1.119 (.160)
Block 3	1.209 (.128)†	1.240 (.175)
Block 4	1.360 (.204)*	1.409 (.283)†
Block 6	.955 (.162)	.956 (.215)
Block 7	1.185 (.209)	1.231 (.286)
Block 9	.513 (.096)***	.712 (.169)
Block 10	.869 (.168)	1.002 (.260)
Block 11	1.205 (.237)	2.082 (.520)**
Constant	3.509 (.478)	1.827 (.337)
/lnalpha	-3.080 (.291)	-2.413 (.275)
Alpha	.046 (.013)	.090 (.025)

Likelihood-ratio test of alpha = 0 for pre/during intervention model: χ^2(df=1) = 53.86, p < .0001
Likelihood-ratio test of alpha = 0 for pre/post intervention model: χ^2(df=1)= 77.57, p < .0001

Table S40: Negative Binomial Regression Models for Total Citizen-Initiated Crime Calls

This table presents the full negative binomial regression models that are shown in Table 3 and Table 4 in the main paper. 120 hot spots included in the analysis. The columns present the IRR (standard error). P-values are two-tailed tests: † $p < .10$. * $p < .05$. ** $p < .01$. *** $p < .001$. The pre-intervention crime counts were logged values. Counts of 0 were changed to 0.5, for a logged value of -0.69.

Variable	*Total Citizen-initiated crime calls pre/during intervention*	*Total Citizen-initiated crime calls pre/post intervention*
Pre-Intervention Crime	2.018 (.085)***	2.175 (.100)***
Group	.908 (.068)	.949 (.083)
Cambridge	.505 (.094)***	.204 (.043)***
Houston	.744 (.122)†	.286 (.054)***
Block 2	.973 (.150)	.954 (.165)
Block 3	1.091 (.163)	1.074 (.180)
Block 4	1.458 (.308)†	1.318 (.313)
Block 6	1.254 (.220)	.724 (.157)
Block 7	1.796 (.339)**	1.269 (.280)
Block 9	.994 (.165)	1.008 (.203)
Block 10	1.021 (.201)	.962 (.221)
Block 11	1.489 (.315)†	1.836 (.439)*
Constant	5.184 (1.027)	7.257 (1.588)
/lnalpha	-2.116 (.192)	-1.831 (.216)
Alpha	.120 (.023)	.160 (.035)

Likelihood-ratio test of alpha = 0 for pre/during intervention model: χ^2(df=1) = 260.55, p < .0001
Likelihood-ratio test of alpha = 0 for pre/post intervention model: χ^2(df=1) = 265.66, p < .0001

Table S41: Negative Binomial Regression Main Effect Results for Arrests by Officer during the Intervention Period with Houston Data Omitted

The results column reports the IRR (standard error). The p-value reflects two-tailed tests: † p < .10. * p < .05. ** p < .01. *** p < .001. Below the table, the likelihood-ratio test that alpha equals zero, which compares this model to a Poisson model. The results strongly suggest that the alpha is non-zero and the negative binomial model is more appropriate than the Poisson model.

Variable	*Tucson & Cambridge only (n=20) IRR (Std. Error)*
Group	.448 (.142)*
Pre-intervention arrests (Ln)	1.858 (.467)*
Cambridge	.138 (.119)*
Constant	5.187 (5.302)
Ln(alpha)	-1.631 (.610)
Alpha	.196 (.119)

Likelihood-ratio test of alpha = 0: χ^2(df=1) =33.74, p < .0001

Table S42: Community Survey Findings for Procedural Justice, Legitimacy, and Police Misbehavior with Houston Data Omitted

Means based on margins calculated in ANOVA models for each outcome. N represents the number of hot spots. Cohen's *d* calculated based on $d = (M_2 - M_1) / SD_{pooled}$. One-tailed p-value for F test for group effect.

Outcome (n)	*Procedural Justice Post-Pre Mean(Std. dev.)*	*Standard Cond. Post-Pre Mean(Std. dev.)*	*Cohen's d*	*F (num. df, denom. df)*	*p-value*
Procedural Justice and Legitimacy					
Procedural justice (80)	-.005 (.209)	-.015 (.323)	0.038	.03 (1,72)	.434
Legitimacy on the block (80)	-.049 (.153)	.025 (.241)	-0.118	2.71 (1,72)	.052
Legitimacy citywide (80)	.004 (.207)	-.009 (.260)	0.058	.07 (1,72)	.399
Police Misbehavior					
Police harass or mistreat (80)	-.046 (.275)	.077 (.240)	-0.479	4.84 (1,72)	.016
Police use too much force (80)	-.046 (.301)	.037 (.368)	-0.246	1.17 (1,72)	.142

Table S43: Negative Binomial Regression Models for Total Crime Incidents and Total Crime Calls Pre/During and Pre/Post Intervention with Houston Data Omitted

80 hot spots included in the analysis. The columns present the IRR (standard error). P-values are two-tailed tests: † p < .10. * p < .05. ** p < .01. *** p < .001. The pre-intervention crime counts were logged values. Counts of 0 were changed to 0.5, for a logged value of -0.69.

Variable	*Total crime incidents pre/during intervention*	*Total crime incidents pre/post intervention*	*Total citizen-initiated crime calls pre/during intervention*	*Total citizen-initiated crime calls pre/post intervention*
Pre-Intervention Crime	2.056 (.139)***	2.160 (.198)***	2.289 (.115)***	2.446 (.114)***
Group	.903 (.061)	.851 (.081)†	.900 (.069)	.983 (.073)
Cambridge	.707 (.149)	.668 (.189)	.654 (.114)*	.257 (.044)***
Block 2	1.213 (.128)†	1.127 (.167)	.995 (.126)	.973 (.111)
Block 3	1.212 (.127)†	1.201 (.177)	1.049 (.127)	1.038 (.113)
Block 4	1.372 (.213)*	1.240 (.273)	1.325 (.226)†	1.215 (.188)
Block 6	.975 (.167)	.918 (.213)	1.143 (.179)	.663 (.120)*
Block 7	1.248 (.237)	1.048 (.271)	1.441 (.250)*	1.039 (.187)
Constant	3.936 (.973)	2.384 (.793)	3.192 (.707)	4.523 (.929)
/lnalpha	-3.111 (.314)	-2.331 (.290)	-2.617 (.243)	-2.768 (.282)
Alpha	.045 (.014)	.097 (.028)	.073 (.018)	.063 (.018)

Likelihood-ratio test of alpha = 0 for incidents pre/during intervention model: χ^2(df=1) = 47.97, p < .0001
Likelihood-ratio test of alpha = 0 for incidents pre/post intervention model: χ^2(df=1) = 75.24, p < .0001
Likelihood-ratio test of alpha = 0 for crime calls pre/during intervention model: χ^2(df=1) = 142.05, p < .0001
Likelihood-ratio test of alpha = 0 for crime calls pre/post intervention model: χ^2(df=1) = 177.33, p < .0001

References

1. W. G. Skogan, M. Van Craen, C. Hennessy, Training police for procedural justice. *J. Exp. Criminol.* **11**, 319-334 (2015).
2. L. Wheller, P. Quinton, A. Fildes, A. Mills, “The Greater Manchester Police procedural justice training experiment,” (College of Policing, Coventry, 2013).
3. N La Vigne, J. Jannetta, J. Fontaine, D. S. Lawrence, S. Esthappan, “The National Initiative for Building Community Trust and Justice: Key process and outcome evaluation findings,” (Urban Institute, Washington, DC, 2019).
4. R. S. Engel, N. Corsaro, G. T. Isaza, H. D. McManus, “Examining the impact of *Integrating Communications, Assessment, and Tactics (ICAT)* de-escalation training for the Louisville metro Police Department: Initial Findings,” (University of Cincinnati, 2020).
5. D. P. Rosenbaum, D. S. Lawrence, Teaching procedural justice and communication skills during police-community encounters: Results of a randomized control trial with police recruits, *J. Exp. Criminol*. **13**, 293-319 (2017).
6. S. D. Mastrofski, R. B. Parks, J. D. McCluskey, “Systematic social observation in criminology” in *Handbook of Quantitative Criminology,* A. R. Piquero, D. Weisburd, Eds. (Springer, 2010), chap. 12.
7. T. Jonathan-Zamir, S. D. Mastrofski, S. Moyal, Measuring procedural justice in police-citizen encounters. *Justice Q*. **32**, 845-871 (2015).
8. A. Diamantopoulos, H.M. Winklhofer, Index construction with formative indicators: An alternative to scale development. *J. Marketing Research*, **32**, 269-277 (2001).
9. S. B. McKenzie, P.M. Podsakoff, & C. B. Jarvis, The problem of measurement model misspecification in behavioral and organization research and some recommended solutions. *J. Applied Psych.* **90**, 710-730 (2005).
10. Williams, R. Genearlized ordered logit/partial propotional odds models for ordinal dependent variables. *The Stata J.* **6**, 58-82 (2006).
